# Snapshots of a solid-state transformation: coexistence of three phases trapped in one crystal†
†Electronic supplementary information (ESI) available: X-Ray crystallography, DSC, optical microscopy and Raman spectroscopy experimental details; crystallographic tables and figures; TOPOs diagrams; *F*_o_–*F*_c_ plots; powder-X diffractograms; magnetic susceptibility; Raman and optical microscopy. CCDC 1053051–1053059. For ESI and crystallographic data in CIF or other electronic format see DOI: 10.1039/c5sc04287a


**DOI:** 10.1039/c5sc04287a

**Published:** 2016-01-05

**Authors:** G. Aromí, C. M. Beavers, J. Sánchez Costa, G. A. Craig, G. Mínguez Espallargas, A. Orera, O. Roubeau

**Affiliations:** a Departament de Química Inorgànica , Universitat de Barcelona , Diagonal 645 , 08028 Barcelona , Spain . Email: guillem.aromi@qi.ub.es; b Advanced Light Source , Berkeley Laboratory , 1 Cyclotron Road , Berkeley , CA 94720 , USA . Email: cmbeavers@lbl.gov; c Instituto de Ciencia Molecular (ICMol) , Universidad de Valencia , c/ Catedrático José Beltrán, 2 , 46980 Paterna , Spain; d Instituto de Ciencia de Materiales de Aragón (ICMA) , CSIC and Universidad de Zaragoza , Plaza San Francisco s/n , 50009 , Zaragoza , Spain . Email: roubeau@unizar.es

## Abstract

Crystal-to-crystal transformations have been crucial in the understanding of solid-state processes, since these may be studied in detail by means of single crystal X-ray diffraction (SCXRD) techniques. The description of the mechanisms and potential intermediates of those processes remains very challenging. In fact, solid-state transient states have rarely been observed, at least to a sufficient level of detail. We have investigated the process of guest extrusion from the non-porous molecular material [Fe(bpp)(H_2_L)](ClO_4_)_2_·1.5C_3_H_6_O (bpp = 2,6-bis(pyrazol-3-yl)pyridine; H_2_L = 2,6-bis(5-(2-methoxyphenyl)-pyrazol-3-yl)pyridine; C_3_H_6_O = acetone), which occurs through ordered diffusion of acetone in a crystal-to-crystal manner, leading to dramatic structural changes. The slow kinetics of the transition allows thermal trapping of the system at various intermediate stages. The transiting single crystal can be then examined at these points through synchrotron SCXRD, offering a window upon the mechanism of the transformation at the molecular scale. These experiments have unveiled the development of an ordered intermediate phase, distinct from the initial and the final states, coexisting as the process advances with either of these two phases or, at a certain moment with both of them. The new intermediate phase has been structurally characterized in full detail by SCXRD, providing insights into the mechanism of this diffusion triggered solid-state phenomenon. The process has been also followed by calorimetry, optical microscopy, local Raman spectroscopy and powder X-ray diffraction. The discovery and description of an intermediate ordered state in a molecular solid-state transformation is of great interest and will help to understand the mechanistic details and reaction pathways underlying these transformations.

## Abstract 2

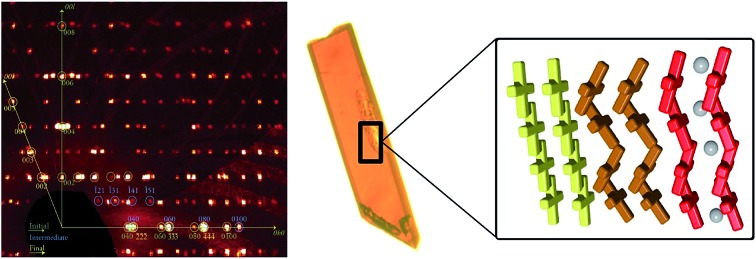
Solvent extrusion leads to crystallographic–magnetic transition within a molecular complex *via* an intermediate that can be trapped and characterized.

## Introduction

Crystal-to-crystal processes constitute an increasingly important area of materials science. When a solid-state transformation occurs between two such ordered states, single crystal X-ray diffraction (SCXRD) techniques open the window to a microscopic landscape of invaluable information on these phenomena. Crystallographic phase transitions resulting from the occurrence of a novel thermodynamic minimum require an external stimulus, which may or may not lead to variations in the chemical composition.^1^ Paradigmatic examples of the former case are some crystal-to-crystal changes following a photo-isomerization phenomenon, such as those leading to photomechanical actuators.^2^,^3^ Entropy driven transformations resulting from temperature changes often fall into this category as well.^4^–^7^ Chemical changes may result from exposure to gases,^8^–^12^ liquids^13^–^15^ or solutes^15^–^17^ prompting their absorption or their exchange by other guest molecules, or from evacuation of hosted molecules following “evaporation”,^11^,^13^ temperature increases^8^,^12^,^18^,^19^ or depressurization.^10^ The transiting species are usually involved in weak intermolecular interactions with the host, but they can also form coordination bonds^16^,^18^,^20^ or even covalent interactions with it,^21^ leading to major structural changes which can be depicted by SCXRD. Indeed, modifications of true chemical bonds in the context of crystal-to-crystal processes have led to the emerging area in solid-state chemistry concerned with the concept of post-synthetic modification (PSM)^22^ of metal–organic frameworks (MOFs). Sometimes, the host acts simply as the cavity where chemical reactions of guests occur, which can be in this manner examined by SCXRD.^23^–^25^ Similarly, SCXRD has been suggested as an analytical tool on the nanogram scale for the structural determination of exotic molecules incorporated inside the lattice of a single crystal,^26^ through a method that is in the process of finally being applicable.^27^ Despite these groundbreaking achievements, the elucidation of the mechanism by which solid-state transformations occur using conventional SCXRD still remains a huge challenge.^14^ This requires the process to occur through a succession of ordered states, including the potential presence of intermediate phases, and the capacity to “freeze” or trap them kinetically for structural determination. At a molecular level, it has been possible to trap discrete intermediates for the SCXRD elucidation of their structure during chemical transformation or movement of molecules within a crystalline host.^23^–^25^ However, such description of metastable intermediate phases for the extended lattice itself has never been achieved. In an exceptional case, a first order crystallographic crystal-to-crystal transition of a non-porous molecular solid following a guest inclusion process could be stopped at an intermediate stage and the structure of the system analyzed.^14^ The results showed the initial and the final ordered phases to both coexist within the same single crystal. More recently, a porous system suitable for guest-exchange was designed to slow down the diffusion process by employing guest molecules of a size very similar to the MOF's channel dimensions.^28^ It was then possible to identify the formation at the surface of an ordered *transient state* of the framework by means of grazing incident X-ray diffraction and obtain a very approximate picture of its structure. It is worth mentioning as well the seminal crystallographic studies of photo-induced metastable intermediates of several types of photochemical transformations.^29^,^30^

**Fig. 1:**
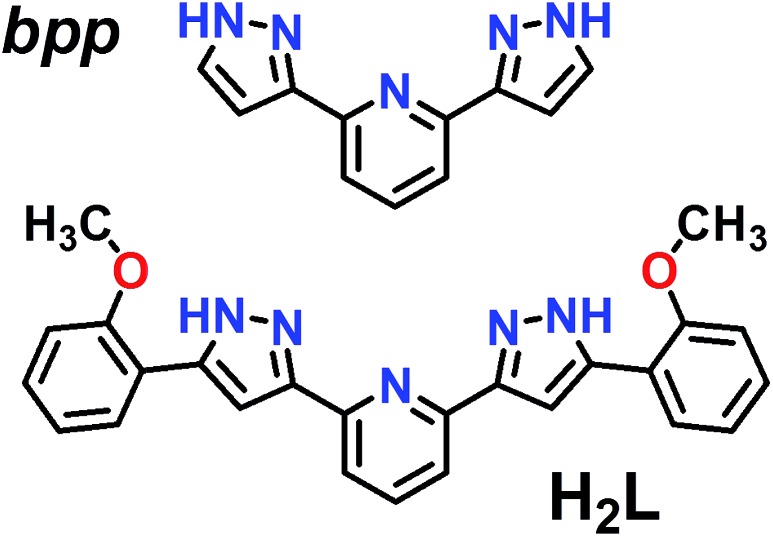
Representation of ligands bpp (2,6-bis(pyrazol-3-yl)pyridine) and H_2_L (2,6-bis(5-(2-methoxyphenyl)-pyrazol-3-yl)pyridine).

We report here the use of SCXRD to monitor the remarkable crystal-to-crystal transformation of a non-porous molecular lattice involving the diffusion of acetone molecules, a process which has been quenched at various stages providing direct structural evidence of the mechanism of guest evacuation. Single crystals of [Fe(bpp)(H_2_L)](ClO_4_)_2_·*x*C_3_H_6_O (**1**) (bpp = 2,6-bis(pyrazol-3-yl)pyridine; H_2_L = 2,6-bis(5-(2-methoxyphenyl)-pyrazol-3-yl)pyridine; C_3_H_6_O = acetone, Fig. 1) had been shown to exhibit reversible and robust absorption and desorption of acetone, accompanied by dramatic changes to the structural, magnetic and optical properties.^31^ We have now been able to freeze the process of diffusion of acetone removal at various stages and take “snapshots” of the transformation by SCXRD. This has led to the discovery and full crystallographic description of an ordered metastable transient phase of the lattice, different from the initial and final ones. While intermediate states of a dehydration process have been indeed characterized before, these are indeed stable and isolable;^32^ however, characterizing a pure metastable phase crystallographically represents a finding never seen before in the context of solid-state transformations. Astonishingly, these three phases coexist during certain stages of the transformation within a single crystal, and the three could be refined simultaneously from the same data set, thus providing direct structural evidence of the reaction pathway. This has opened an unprecedented window to the mechanistic studies of concerted solid-state phase transformations. Such discovery is not only relevant to the areas such as spin crossover or coordination chemistry, but to all the scientific domains dealing with solid state transformations in the crystalline form.

## Results and discussion

### Single crystal X-ray diffraction

**Fig. 2:**
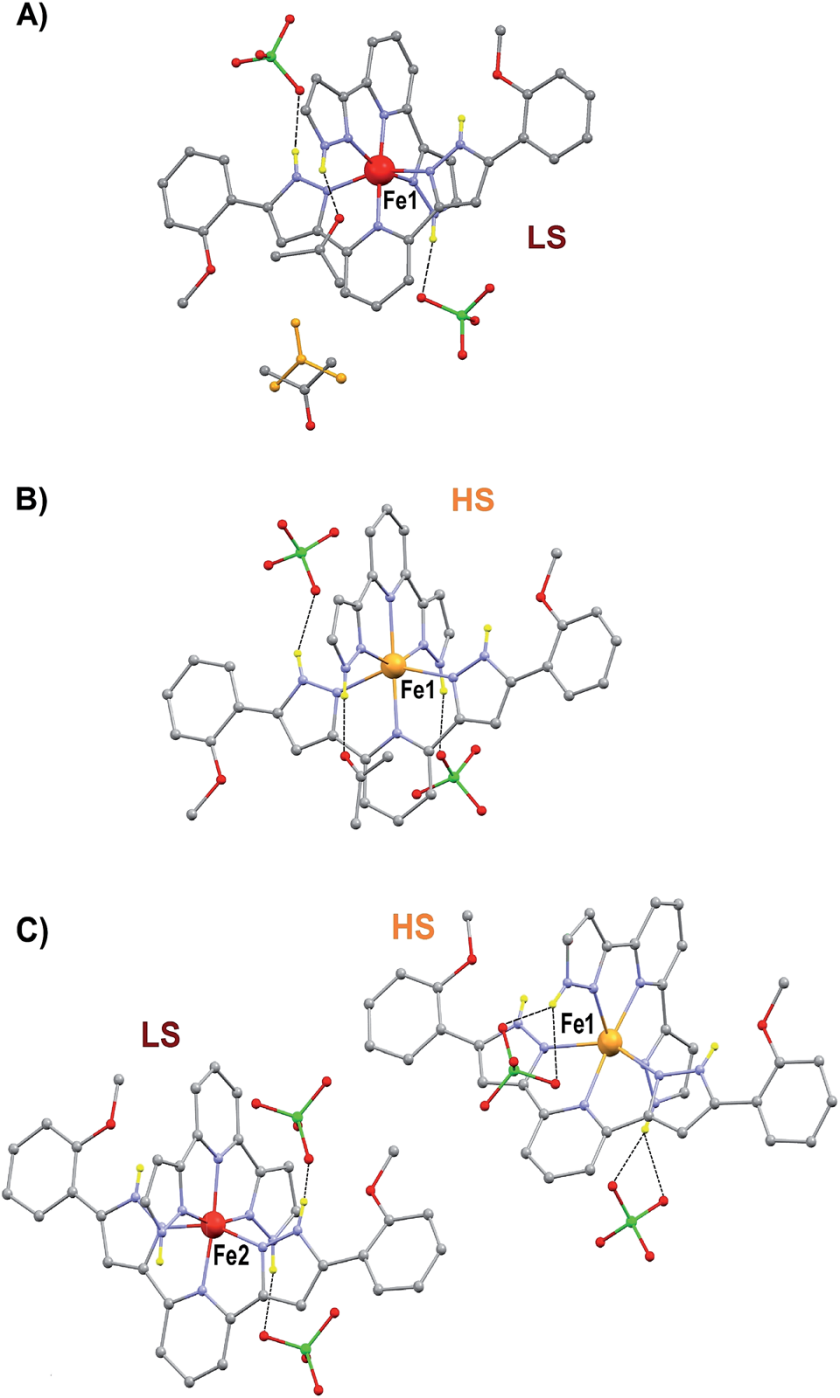
Representation of the molecular units of the different solvatomorphic phases of [Fe(bpp)(H_2_L)](ClO_4_)_2_ (**1**); **1i** (A), **1f** (B) and **1t** (C). Grey, C; red, O; green, Cl; purple, N; red, Fe(ii) LS; orange, Fe(ii) HS. Only hydrogen atoms on N–H groups shown in yellow. Hydrogen bonds shown with dashed lines. Both positions of the disordered acetone of molecule in **1i** shown (one in yellow).

The preparation and structure of the heteroleptic complex [Fe(bpp)(H_2_L)](ClO_4_)_2_·1.5C_3_H_6_O (1initial; **1i**) was previously described in detail.^31^ It belongs to the *P*2_1_/*n* space group (*V* = 4205.0(9) Å^3^) and contains a cationic unit formed by two different tridentate ligands disposed perpendicular to each other around the central Fe(ii) metal, thus providing a pseudo-octahedral coordination geometry to it (Fig. 2A). The Fe–N distances average 1.949 Å at 250 K, indicating the metal to be in the low spin state (LS; S = 0). The methoxy groups of the distal H_2_L phenyl rings point to opposite directions, the ligand thus exhibiting a *syn*,*anti* configuration. In the lattice, the “cross-shaped” cations interact with each other through complementary donor/acceptor π···π interactions and C–H···π contacts, forming infinite 2D sheets (Fig. S1†). Within these sheets, the methoxyphenyl rings of first- and second-neighbor molecules overlap through π···π contacts leading to infinite stripes of well-stacked six-member rings (Fig. S1†). The cations are disposed in two symmetry related orientations, their idealized equatorial planes forming an angle of 25.61°, which leads to corrugated sheets (Fig. S2†). Two crystallographically independent perchlorate counteranions (both disordered over two very similar positions) and one acetone molecule of crystallization are located in between these sheets, participating as acceptors of three well-defined hydrogen bonds with pyrazolyl N–H groups from the complex cation [Fe(bpp)(H_2_L)]^2+^ (Fig. 2A). There is an additional fraction of 0.5 molecules of acetone (disordered over two positions related by a center of symmetry) in between planes that is held by weak van der Waals interactions. Adjacent sheets of cations feature a crystallographic separation of 10.785 Å, leading to an efficient occupation of the space. Compound **1i** was previously reported to evacuate one half equivalent of acetone upon constant heating at 390 K for 2 h, thus producing the compound [Fe(bpp)(H_2_L)](ClO_4_)_2_·C_3_H_6_O (1final; **1f**) after a crystal-to-crystal transformation that causes a colour change from dark red to orange.^31^ The new compound (Fig. 2B) exhibits dramatic structural differences with respect to **1i**; it features now a *P*1[combining macron] space group, a unit cell reduced approximately by one half (*V* = 2130.8(2) Å^3^), and the H_2_L methoxyphenyl groups of half of the cations have rotated nearly 180° (Fig. S3†), but still disposed as infinite arrays of well packed rings contained within the sheets (Fig. S4†). In addition, all cations are now disposed in the same spatial orientation, leading to non-corrugated sheets (Fig. S5†), which lie slightly closer to each other (10.014 Å). Also, the average of the Fe–N distances (2.169 Å) has increased by 10%, thus indicating that the Fe(ii) ions have experienced a process of spin crossover (SCO),^33^,^34^ now exhibiting the high spin state (HS; S = 2) at 250 K, consistent with the observed colour change. The evacuation of this fraction of acetone molecules was found to be fully reversible upon exposure of the **1f** crystals to vapours of this solvent. The absence of pores in both structures suggests that the 0.5 equivalents of acetone evacuating and re-entering the solid must move by a relatively slow diffusion and organized process throughout the lattice. This system could thus be suited for the monitoring of an exchange process through SCXRD and unveil the mechanism of the transformation, by freezing and studying it at different stages. A single crystal of compound **1i** was cooled down with a cold-stream of gaseous N_2_ from room temperature to 250 K at the goniometer of a diffractometer revealing the structure to be perfectly reproducible and identical to that previously characterized in the same conditions.^31^ The crystal was allowed to warm up to 350 K at a pace of 6 K min^–1^ and was then kept at that temperature during 15 min, before being brought back to 250 K to perform a subsequent SCXRD experiment. The images of the reciprocal space were found to contain diffraction patterns from two samples indicating that the crystal was composed of two different single phases (Fig. S6†). In order to determine the nature of each phase, both groups of reflections were separated by setting up the appropriate sample mask through the SAINT^35^ integration engine (see ESI for details†). It was then possible to solve the structures for both components of the same crystal, at this stage of its transformation (stage 1). The first one corresponds to the lattice of the initial compound **1i**, *i.e.* the original system in the LS state, with the full load of acetone (1.5 equivalents). Most surprisingly, the second structure corresponds to neither **1i** nor **1f**, but it is a completely different phase, **1t** (1transient). This means that in this crystal, the transformation **1i** → **1f** passes through a distinct intermediate phase, which has now been detected and identified. The structure of **1t** belongs to the monoclinic space group *P*2_1_/*c* (*V* = 7805(2) Å^3^) and could only be attributed the formulation [Fe(bpp)(H_2_L)](ClO_4_)_2_. The position and amount of the necessarily present acetone molecules in this transient intermediate state between **1i** and **1f** could not be defined from the scattering data. This is either because they are so disordered that their contribution cannot be detected in the Bragg scattering, perhaps producing just diffuse scattering, or because these molecules could only be represented in modulated intensity, since the presence of satellite reflections on the reciprocal space images (Fig. S6†) might be related to some level of modulation in the structure (see ESI†). No direct evidence from diffuse Bragg scattering was however detected, and in fact a number of potential satellites are likely arising from other phase(s) present at different depth(s) in the reciprocal space.

**Fig. 3:**
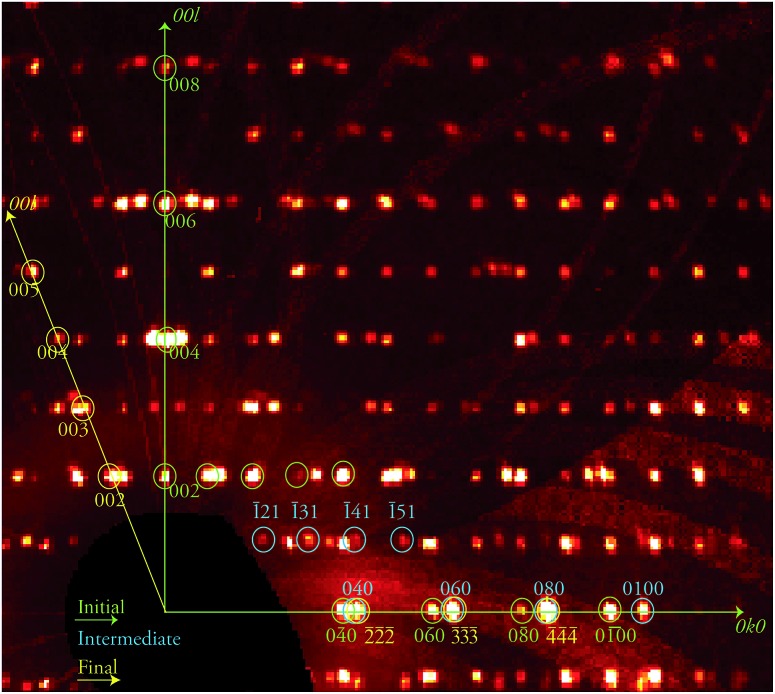
Portion of the reciprocal space pseudo-precession view corresponding to the 0*kl* layer of phase **1i**, produced from the stage 2 data collection and containing patterns from the three phases **1i**, **1t** and **1f**. A selection of the corresponding reflections are shown and labelled in green, blue and yellow, respectively, while the 0*k*0 and 00*l* directions of phase **1i** are shown as green arrows and the 00*l* direction of phase **1f** as yellow arrow, as indicated.

The discovery of a transient, ordered crystallographic phase formed during the course of a crystal-to-crystal transformation and its characterization through SCXRD (see below), in coexistence with the initial phase, is remarkable. In view of this, attempts to trap other combinations of the various phases involved in this process were conducted. Thus, the biphasic crystal just studied was warmed again at the same rate to 370 K and kept at that temperature for another 30 min, before freezing it back to 250 K. An X-ray diffraction experiment was performed anew on the same specimen, now at a further stage of its transformation (stage 2). The result was again an extremely complicated diffraction pattern (Fig. 3) which corresponds to three different phases. This complex data set was successfully solved as a combination of the three known phases **1i**, **1t** and, **1f** respectively, proving the coexistence of these three ordered lattices within a crystal at this one point of the **1i** → **1f** transformation. This transformation can be allowed to proceed further in a controlled manner by submitting the crystal to the same treatment as in the previous experiment now maintaining a high temperature plateau for a longer period. A novel diffraction experiment (stage 3) produced a new pattern now composed of two ordered independent phases that were solved for **1f**, the final phase of the process, and **1t**, the intermediate, with a complete absence of **1i**.

**Fig. 4:**
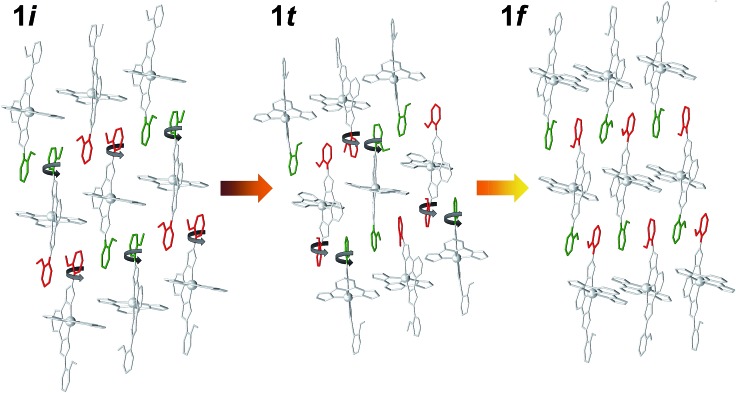
Representation of [Fe(bpp)(H_2_L)]^2+^ cations in **1i**, **1t** and **1f**, emphasizing the rotation by 180° of 50% of the distal methoxyphenyl groups of H_2_L in passing from one to the other. Green and red colors represent the two orientations of the methoxyphenyl groups with respect to the plane of the sheet.

The structure of **1t** could be refined from the three data sets where it was identified: **1i** + **1t** (stage 1), **1i** + **1t** + **1f** (stage 2, used for the description below) and **1t** + **1f** (stage 3). Its asymmetric unit contains two crystallographically independent ionic ensembles of [Fe(bpp)(H_2_L)](ClO_4_)_2_ (Fig. 2C). The [Fe(bpp)(H_2_L)]^2+^ cations share the identity with these from **1i** and **1f**, with the remarkable difference that **1t** possesses half of the distal methoxyphenyl groups rotated by nearly 180° with respect to any of the other two isomers. Thus, the ligands H_2_L exhibit a *syn*,*syn* conformation. Also noticeably, one of the two different complex cations of **1t** is in the LS state at 250 K (Fe1; *avg* Fe–N, 1.954 Å) whereas the other features a HS state (Fe2; *avg* Fe–N, 2.158 Å). Thus, the transient phase consists, at 250 K, of a mixed HS/LS system with ordered magnetic structure. Each complex cation interacts with four nearest neighbors and two second-nearest neighbors through a total of six π···π and eight CH···π interactions (Fig. S7†) forming infinite sheets that stack parallel to each other. Within these sheets, one can observe again the linear succession of methoxyphenyl rings connected to each other forming infinite stripes. In such organization, arrays of HS cations alternate with lines of LS compounds along the crystallographic *c* axis, whereas the *a* direction within the sheets features chains of cations alternating the HS and LS states (Fig. S7†). In this transient state, each independent complex cation exhibits two spatial orientations, with angles between idealized equatorial planes of 30.93°, 38.72°, 41.99° and 57.38°, which leads to a very high degree of corrugation of the sheets where they reside (Fig. S8†). In addition, three of the four independent ClO_4_^–^ groups are disordered. As in **1i** and **1f**, these anions form hydrogen bonds with N–H groups from the H_2_L ligands, with the difference that each cation of **1t** interacts with three anions instead of two, thus implying that two of the independent ClO_4_^–^ species in turn interact simultaneously with two [Fe(bpp)(H_2_L)]^2+^ complexes. As a result, the N–H group that holds one molecule of acetone through a hydrogen bond in **1i** and **1f** is here taken by a ClO_4_^–^ anion. Because of this and given the conformation (*syn*,*syn*) of the H_2_L ligands, none of the N–H groups are available for acetone molecules to establish hydrogen bonds with any pyrazolyl from the complex. Therefore, this solvent is now probably distributed in a highly disordered manner in between the sheets, perhaps silent to the X-ray diffraction experiment and presumably exhibiting high mobility. The arrangement in **1t** causes the layers of [Fe(bpp)(H_2_L)]^2+^ cations to pack slightly closer to each other than within the other two phases (Fig. S8;† separation of 9.269 Å). This set of experiments demonstrates that the process of evacuation of one half equivalent of acetone from **1i** occurs in an organized manner, presumably starting at the surface of the crystal (*vide infra*) with the growth of a well-defined and crystallographically distinct transient state (**1t**). Most likely, the amount of acetone contained within **1t** is variable, which in part could explain the fact that no ordered acetone is detected. In fact, a crucial question to elucidate with regard to this system is the mechanism by which the diffusion of the solvent molecules takes place and how it leads to a complete crystal-to-crystal transformation that is reversible. Inspection of the three lattices participating in this process shows an aspect that seems to be paramount: in passing from **1i** to **1t** half of the methoxyphenyl rings of the H_2_L ligands (one group per ligand) rotate by nearly 180° (Fig. 4). It is very likely that this rotation occurs in a concerted manner as the front of the interface advances through the material, leading to a highly corrugated lattice. In this respect, it must be noticed that the leaving acetone molecule is closest to the two rotating –OMe groups of both sheets sandwiching it (Fig. 1A and S9†). The transition between the intermediate (**1t**) and the final phase (**1f**), also occurs through the rotation of 50% of the distal phenyl rings in the [Fe(bpp)(H_2_L)]^2+^ cations (Fig. 4), producing a phase composed this time of very flat sheets. An intermediate phase with a variable composition of highly mobile acetone, located in between the surface and initial phase at the interior of the crystal, may be the key to facilitate a complete crystal-to-crystal transformation. The non-detection of acetone in the intermediate structure is an indication of this higher mobility. This would be facilitated by the fact that the conformation of [Fe(bpp)(H_2_L)](ClO_4_)_2_**1** in this phase (**1t**) prevents the establishment of strong H-bonding interactions between C_3_H_6_O molecules and the complex cation (*vide supra*). The successive formation of phases during the pseudomorphic **1i** → **1t** → **1f** transformation is different from previous reports on SCO processes concomitant to phase transitions in that here the process takes place with changes to the composition.^29^,^36^

The program TOPO^37^ was employed to understand the relationships between the coexisting unit cells, applying the methodology of Etter and Gougoutas.^38^,^39^ This program uses the orientation matrices of the samples in order to compare unit cell vectors, and allows determining relationships between the successive phases. Reciprocal lattice vectors 010 and 111[combining macron] of **1i** were found to be nearly parallel to 010 and 01[combining macron]2[combining macron], respectively, of **1t** (Fig. S10†). In the case of the twinned phase **1f**, the two domains are related by the lost 2-fold rotation, which is coincident with the 111 reciprocal axis for both domains. The reciprocal lattice vector 020 in **1t** was found to be very nearly parallel with 1[combining macron]1[combining macron]1[combining macron] of both **1f** domains, and 12[combining macron]3 of the intermediate was found to be nearly parallel to 021 of the first domain and 201 of the second domain of **1f** (Fig. S11†). Overall, the successive transitions **1i** → **1t** → **1f** are thus topotactic,^38^,^39^ while the **1f** phase is a conservative twin, as expected for such transformations. Despite the limitations inherent to data containing diffraction patterns from 2 to 4 crystallographic domains the structural models and refinements are very satisfactory, as reflected in the *F*_o_–*F*_c_ maps for each of the three intermediate stages analyzed, which confirm the good agreement between the structural models and electron density maps (Fig. S12 to S14†).

### Powder X-ray diffraction

**Fig. 5:**
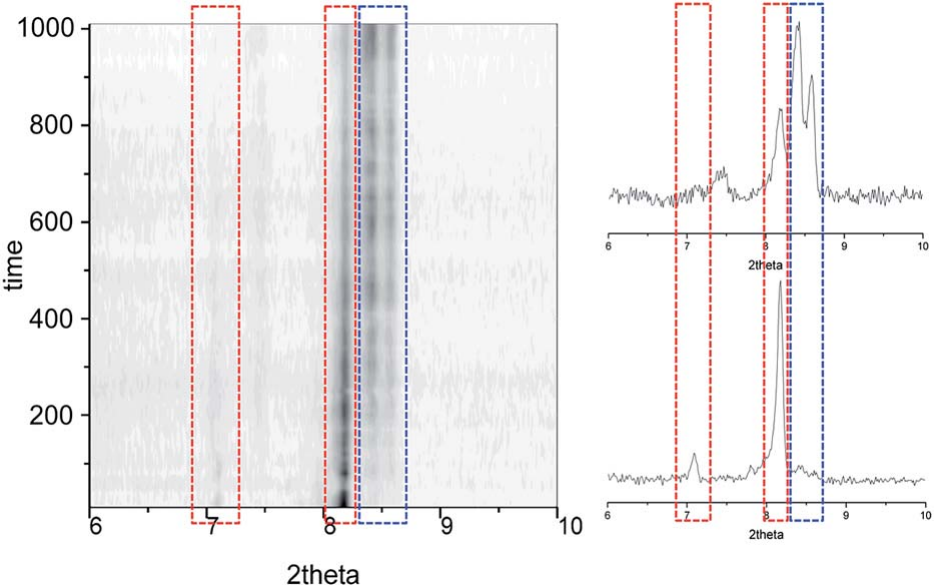
Time dependence of the PXRD diagrams from Pawley refinements of **1i**, performed at 353 K, showing the gradual evolution from **1i** to **1f**, here without the observation of the transient phase.

Further information of the **1i** → **1f** solid-state transformation was obtained from powder X-ray diffraction (PXRD) experiments. In order to perform the experiments and insert a polycrystalline sample of **1i** inside a borosilicate capillary tube it was necessary to fragment the large single crystals to less than one tenth of the size of those of previous experiments. The sample was warmed at 353 K and Pawley refinements were performed every 10 min, showing a gradual evolution from **1i** to **1f** without a trace of the transient phase (Fig. 5 and S15†). This absence of **1t** indicates that a critical size of the initial ordered phase is necessary for any appreciable amount of the transient phase to develop before the conversion to the final state of the process occurs. Such phenomenon could explain why potential transient phases of solid-state transformations have rarely been detected by PXRD.^40^,^41^

### Magnetic susceptibility

**Fig. 6:**
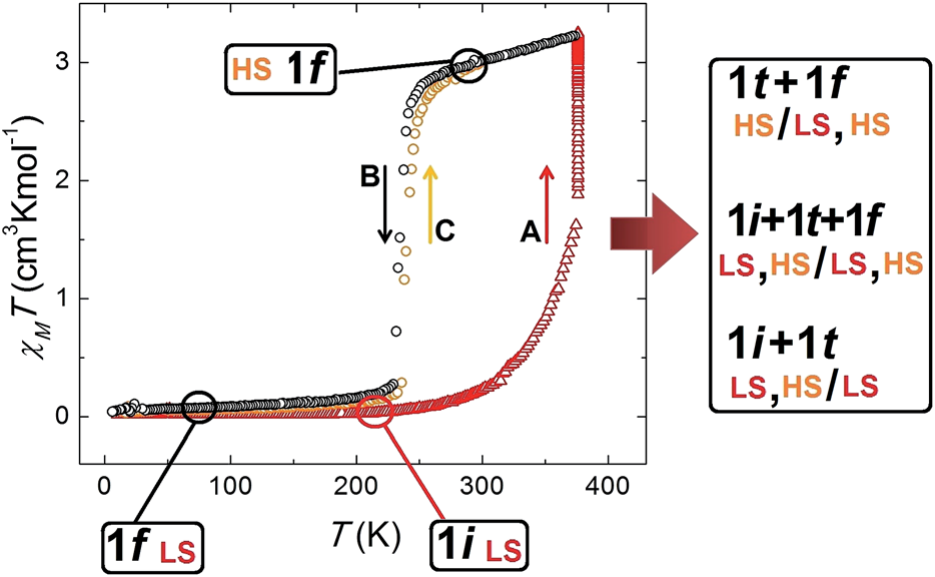
*χT vs. T* plot of **1** throughout the thermal process leading to the release of acetone concomitant to a switch of spin states and then to reversible SCO, which is consistent with the succession of crystallographic phases detected by single crystal X-ray diffraction. Starting with phase **1i** at 2 K (LS, red triangles), heating it to 375 K and maintaining the temperature (segment A), the value of *χT* starts to increase as the phase transformation **1i** → **1t** → **1f** proceeds with the solvent desorption. Once this process is finalized, the product, **1f**, is in the HS state (black circles). Upon cooling, a SCO of **1f** is observed (segment B), consistent with the structure at 150 K (LS). This process is reversible, as seen when warming this phase again (segment C, orange circles).

The fact that this process is coupled to one of SCO allows the monitoring by magnetic susceptibility measurements (Fig. 6). The associated colour change from dark red to light orange is also useful to follow the transformation by optical microscopy observation (see below). Thus the *χT* (*χ*; molar paramagnetic susceptibility) product of **1i** shows a diamagnetic behaviour typical of Fe(ii) centres in the LS. This value increases with warming beyond room temperature and then when *T* stays constant at 375 K. This corresponds to the transformation **1i** → **1t** → **1f**. The value of *χT* reaches saturation near 3 cm^3^ K mol^–1^ in about 50 min (Fig. S16†) and then remains stable, consistent with the HS state. If the temperature is lowered again, the system remains in the HS state until near 240 K, when it experiences a process of reversible SCO, featuring a small hysteresis 5 K wide (Fig. 6).^31^ The structure of **1f** in the LS state was determined at 150 K and remains unchanged (Fig. S17†) from the original one except for the expected changes to the Fe–N bond distances derived from the SCO (see Table S4†).

### Differential scanning calorimetry (DSC)

**Fig. 7:**
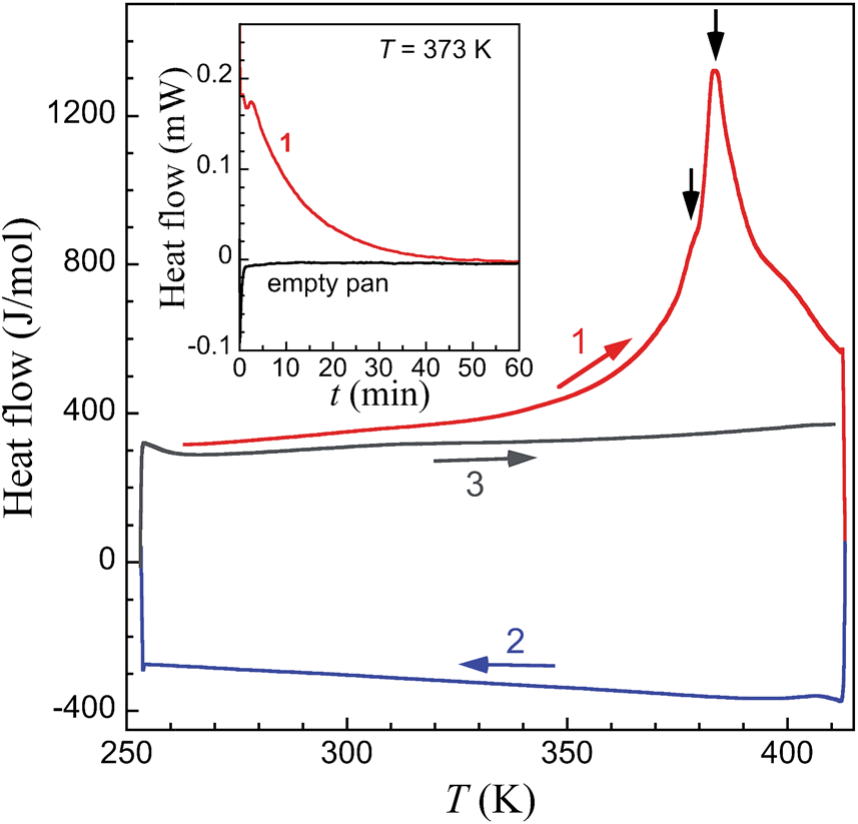
DSC traces of fresh crystals of **1i** at variable temperature in the range 260 to 408 K. After a first warming scan (1, red line) exhibiting a broad endothermic anomaly with two changes of slope on top of this broad anomaly (black arrows), subsequent cooling (2, blue line) and warming (3, grey line) scans are featureless. The inset shows the DSC isotherm trace of fresh crystals of **1i** at *T* = 373 K (red line), compared with the traces obtained under the exact same conditions for an empty Al pan (black line).

DSC measurements on large crystals of **1i** (Fig. 7) support the unique structural observations made here and provide a thermal signature for the various underlying processes: evaporation of acetone, structural phase transitions and associated spin state changes. Indeed a broad endothermic anomaly centred at 383 K is observed over the 353–408 K temperature range in the first warming scan, and can be ascribed to the progressive vaporization of acetone. Two changes of slope on top of this broad anomaly (black arrows in Fig. 6) are observed that could correspond to the successive transformations **1i** → **1t** and **1t** → **1f**, the latter process in the range 380–390 K being clearly sharper. The total enthalpy involved, of 38.8 kJ mol^–1^, accounts reasonably well for the sum of the vaporization of half a molecule of acetone (the heat of vaporization of acetone at 345 K is 28.1 kJ mol^–1^),^42^ the electronic contribution due to the SCO and the structural modifications described.‡
‡A good reference for this can be obtained from the related homoleptic compound [Fe(H_4_L)_2_](ClO_4_)_2_·2acetone·H_2_O (see ref. 5), to which was attributed an enthalpy change of 9.3 kJ mol^–1^ for the SCO and associated order–disorder structural transition. As the latter occurs at lower temperatures and the structural modifications are less important, this value should only be taken as a rough under-estimation of the corresponding enthalpy in the transformation of **1i** to **1t** to **1f**. Subsequent cooling and warming scans are featureless indicating that the transformation **1i** to **1f** is completed after the first warming scan. Isothermal DSC trace at 373 K, *i.e.* close to the first slope change observed in the variable temperature traces, depicts a decreasing endothermic process until *ca.* 60 min, the content of which can be ascribed to the progressive loss of acetone and associated transformation **1i** to **1f**.

### Optical microscopy

**Fig. 8:**
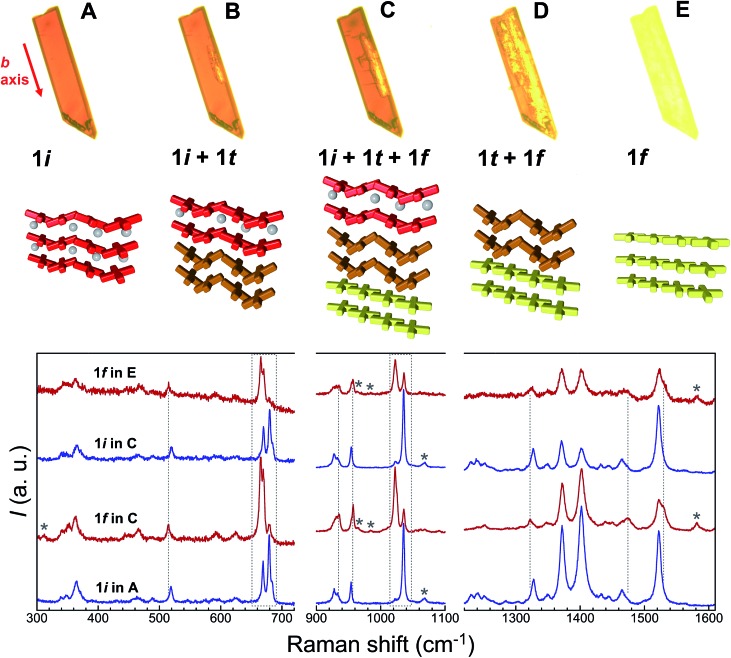
Top: transmission optical microscopy images taken on a single crystal of **1i** at 323 K (A), then passed 4 min at 378 K (B), after being cooled back to 323 K (C), and finally at 387 K (D), during the subsequent warming process up to 413 K (E). These images correspond, approximately, to the different stages of the crystal-to-crystal transformation of **1i** upon acetone evacuation, namely to **1i**, (**1i** + **1t**), (**1i** + **1t** + **1f**), (**1t** + **1f**) and **1f**, respectively, as represented below in form of cartoons. The red arrow indicates the monoclinic *b* axis of **1i**. Bottom: characteristic ambient temperature Raman spectra of the remaining red and orange-yellow areas of partially transformed crystals compared with those of the initial **1i** and transformed **1f** phases. Dashed vertical lines and areas as well as stars highlight modifications characteristic of the **1i** to **1f** transformation (see text).

Additional evidence of the above sequence of phases came from optical microscopy, performed in transmission mode, as well as Raman spectroscopy. Crystals of **1i** were submitted to a thermal history similar to that of the SCXRD studies, namely, warming from room temperature to either 373 or 378 K at a speed of 5 K min^–1^, then holding at these temperatures and cooling rapidly to ambient temperature after varying amounts of time (Fig. 8, S18 and S19†). The crystals originally containing 100% of **1i** are dark red, consistent with the fact that all Fe(ii) centres of this phase are LS. Slightly lighter areas then start to form, apparently from the edges of the crystal, which expand towards the interior as shown by the crystal becoming increasingly orange-yellow. Interestingly, these lighter areas also tend to grow along the elongated axis of the crystals, which was found to be systematically the *b* axis of the monoclinic cell. This suggests that the flux of acetone molecules occurs along this axis, and therefore, perpendicular to the sheets of [Fe(bpp)(H_2_L)]^2+^ cations. At some stage, cooling rapidly freezes the process, leaving the crystal with an unmodified dark red volume coexisting with an orange-yellow phase together with intermediate dark orange areas that can reasonably be ascribed to phases **1i**, **1f** and **1t**, respectively. If phase **1t** still contains 50% of LS metal ions at the temperature of the phase transition, it would be difficult to discern it from **1i** by optical means, as acetone leaves the crystal. However, when the process is sufficiently advanced, the development of phase **1f** is clearly perceived optically, since it shows the orange-yellow colour expected from its composition of all HS Fe(ii), and thus the remaining orange-red areas correspond to any other combination of phases. As the process continues, either by implementing a further plateau at high temperature or by warming continuously the crystal to 413 K, the crystal becomes gradually orange-yellow to its entirety, having transformed to **1f** while maintaining crystallinity, as confirmed by obtaining its characteristic triclinic unit cell.

### Raman spectroscopy

A comparison of the Raman spectra of the various optically-different areas of the intermediate stages with those of fresh **1i** and fully transformed **1f** supports the conclusions obtained by optical observation. Indeed, the characteristic Raman spectra of **1i** and **1f** are obtained when analysing regions of the crystals that appear respectively red, *i.e.* unchanged, or orange-yellow, *i.e.* transformed (Fig. 8). The most striking modifications of the Raman spectra upon the **1i** → **1f** transformation are changes of a group of three bands in the range 660–690 cm^–1^, the reduction of a band at 1034 cm^–1^ in favour of a new band at 1022 cm^–1^, shifts of bands at 518, 953 (likely the symmetric ClO_4_^–^ stretch) and 1326 cm^–1^, the appearance of new bands at 310, 1530 and 1581 cm^–1^ and the disappearance of a band at 1068 cm^–1^. Here it should be noted that, as expected for single-crystal studies, the Raman signatures of the **1i** and **1f** phases vary with the orientation of the crystal studied, especially with respect to relative intensities. However, for each crystal/orientation studied, the coexistence of areas having the characteristic spectra of both, **1i** and **1f** are reproducibly observed along the transformation (see for example Fig. S20†). It should also be stressed that the exact chronology and topology of the transformation for a given thermal cycle strongly depends on the crystal quality; the more defects/cracks present in the crystal the faster the transformation is (see the two crystals studied together in Fig. S19†). Interestingly, when large enough to be analysed, these cracks give a Raman spectrum that, although similar to **1i**, has significantly lower intensity and some slight variations in peak relative intensities and positions (Fig. S21†), and can therefore neither be ascribed to **1i** nor to **1f**. However, the defects/cracks areas cannot be ascribed without uncertainty to **1t** either, since the observed differences may be also due to the reduced crystallinity of these defects and/or the detection of a different crystal orientation. In fact, it is very likely that **1t** only exists beneath the crystal surface, impeding its detection by Raman spectroscopy. Overall, the combination of crystallographic, optical and spectroscopic evidence shows that the transient state coexists sandwiched between the initial and the final phases until the first vanishes at the expense of the transient while the latter then disappears to the benefit of the final form (Fig. 8 and S18†).

## Conclusions

A crystal-to-crystal transformation of the non-porous molecular material [Fe(bpp)(H_2_L)](ClO_4_)_2_·1.5C_3_H_6_O (**1i**) following the evacuation of half equivalent of acetone occurs in an orderly and kinetically slow manner. This allows the thermal quenching of the process at various intermediate stages and its examination through a variety of techniques, most notably by SCXRD. Thus, the process is accompanied by significant structural rearrangements, such as two chronologically separate sequences of rotation of, each time, 50% of all methoxyphenyl rings present in the sample. The transfer of acetone from the bulk of the crystal to the exterior in an ordered manner requires the formation of an intermediate phase, presumably hosting variable and highly mobile acetone, which progressively vanishes to the benefit of the final, evacuated final phase. The identification and full SCXRD characterization of an ordered transient phase during a crystal-to-crystal transformation is fascinating and unprecedented and shall open a new window to the intricate world of dynamic solid-state processes. A fascinating question that warrants further and careful studies is that of the reversibility of the process. Given the established fact that the entirety of the transformations analysed here can be completely reversed also through a crystal to crystal process, it will be of great interest to engage on a study following the various steps through which this reverse process evolves.

## Supplementary Material

Supplementary information

Crystal structure data
