# Supplementary material for: Snapshots of a solid-state transformation: coexistence of three phases trapped in one crystal
Source: Chem Sci. 2016 Jan 5;7(4):2907–15. doi: 10.1039/c5sc04287a (PMC6054035; doi:10.1039/c5sc04287a)
Supplement: Supplementary file 1 [file SC-007-C5SC04287A-s001.pdf]

Electronic Supplementary Material (ESI) for Chemical Science.
This journal is © The Royal Society of Chemistry 2016

Snapshots of a Solid State Transformation: Coexistence of Three Phases Trapped in One Crystal.

Guillem Aromí,*[a] Christine M. Beavers,*[b] José Sánchez Costa,[a] Gavin A. Craig,[a] Guillermo Mínguez Espallargas,[c] Alodia Orera[d] and Olivier Roubeau*[d]

[a] Departament de Química Inorgànica, Universitat de Barcelona, Diagonal 645, 08028 Barcelona, Spain.

[b] Advanced Light Source, Berkeley Laboratory, 1 Cyclotron Road, Berkeley, CA 94720, USA

[c] Instituto de Ciencia Molecular (ICMol), Universidad de Valencia, c/ Catedrático José Beltrán, 2, 46980 Paterna, Spain

[d] Instituto de Ciencia de Materiales de Aragón, CSIC and Universidad de Zaragoza, Plaza San Francisco s/n, 50009, Zaragoza, Spain.

SUPPORTING INFORMATION

Table of contents

Experimental details………………………………………………………………… S2
Crystallographic Tables……………………………………………………………… S6
Figure S1……………………………………………………………………………… S12
Figure S2……………………………………………………………………………… S13
Figure S3……………………………………………………………………………… S13
Figure S4……………………………………………………………………………… S14
Figure S5……………………………………………………………………………… S14
Figure S6……………………………………………………………………………… S15
Figure S7……………………………………………………………………………… S16
Figure S8……………………………………………………………………………… S16
Figure S9……………………………………………………………………………… S17
Figure S10……………………………………………………………………………. S17
Figure S11……………………………………………………………………………. S18
Figure S12……………………………………………………………………………. S19
Figure S13……………………………………………………………………………. S20
Figure S14……………………………………………………………………………. S21
Figure S15……………………………………………………………………………. S22
Figure S16……………………………………………………………………………. S26
Figure S17……………………………………………………………………………. S26
Figure S18……………………………………………………………………………. S27
Figure S19……………………………………………………………………………. S28
Figure S20……………………………………………………………………………. S29
Figure S21……………………………………………………………………………. S30
References…………………………………………………………………………... S31

Experimental details.

Single-crystal X-ray diffraction.

Measurements were performed using a Bruker D8 diffractometer equipped with an APEXII CCD detector on the Advanced Light Source beamline 11.3.1 at Lawrence Berkeley National Laboratory, at λ = 0.7749 Å from a silicon 111 monochromator. The beam size on beamline 11.3.1 is typically 0.250 x 0.200 mm. Several series of datasets were acquired on two different crystals at different stages of the **1*i*** to **1*f*** transformation. For this, a first dark-red crystal of dimensions 0.10 x 0.07 x 0.05 mm^3 (thus completely confined within X-ray the beam) was mounted on a MiTegen loop with little Paraton N grease, mounted on the goniometer in the 250(2) K nitrogen cold stream provided by a Oxford Cryosystems Cryostream 700 Plus, and subjected to various thermal cycles, combining a warming ramp to either 350 or 360 K, an isotherm at this temperature of varying time and a cooling ramp back to 250 K, at which temperature all measurements were done. Additionally, a second dark-red crystal of dimensions 0.50 x 0.40 x 0.30 mm^3 (thus, only partially traversed by the beam) was pre-warmed at 110ºC in an oven so that the transformation would be more advanced, and data were acquired at 293 K. Eventually, data were also acquired at 260 and 100 K on a fully transformed orange crystal of **1*f***, of dimensions 0.18 x 0.14 x 0.08 mm^3, and respectively in its HS and LS states. Besides the initial and final state **1*i*** and **1*f* (HS** and **LS)**, datasets were obtained for situations where a new intermediate phase **1*t*** coexists with either **1*i*** and **1*f*** or both. The images then contain diffraction patterns from two or three samples: the initial LS compound **1*i***, a LS/HS mixed-spin intermediate **1*t*** and the final HS product, **1*f***, which is systematically twinned in these mixtures. The two to four necessary orientation matrices were determined using CELL_NOW and RLATT,[1] and combined into an orientation matrix file (*.p4p). For the initial situation of **1*i*** + **1*t***, reflections were integrated in a standard manner with SAINT[1] as a two-component system. For the **1*i*** + **1*t*** + **1*f*** and **1*t*** + **1*f*** situations, in which the crystals were more damaged, separation and integration of the three or two lists of reflections were done with the SAINT[1] integration engine used in command line mode, and set up with a sample mask. For *each* datasets, the structures of the different phases present were solved and refined by full-matrix least-squares on F^2 with SHELX-TL.[1,2] Details of the treatment of the dataset corresponding to the more complicated case of coexistence of the three phases are given below. Crystallographic and refinement parameters are provided in Tables S1 and S2, respectively for the cases of coexistence of phases and the HS and LS phases of **1*f***.

Sample mask in SAINT:

SMAP=1,2,3,3

This indicates that the first orientation matrix belongs to sample 1, the second belongs to sample 2 and the third and fourth belong to sample 3.

For all commands relating to cell or symmetry later on, comma-delimited lists were used to specify distinct parameters.

Listed below are most of the pertinent SAINT commands:

```
FILTPOINTGP=-1                //all samples integrated as triclinic
CRYST_SYS= 3, 3, 1            //initial-intermediate monoclinic, final triclinic
PAR_MASK= TP, TP, TPS2        //restraining the twinned cells to be similar
GPAR_MASK= TP, TP, TPS2       //also restraining detector pitch and xtal trans
```

The parameter masks are explained in the SAINT manual as "Two-sample integration, where first sample is a two-component twin and second component is a single crystal. In the first sample, constrain cell constants of the second component to those of the first. For both samples, constrain crystal translations and detector pitch"

Unit cell refinement: reflections used and angular range for each sample and component

Sample #	Component #	Reflections	θ_{min}(°)	θ_{max}(°)
Global		3778	2.68	24.64
1	0	1221	2.68	24.56
2	0	1179	2.78	24.64
3	0	1378	3.27	22.69
3	1	688	3.43	22.69
3	2	690	3.27	22.60

Integration statistics for each sample and component

Sample #	Component #	Reflections	$I > 2\sigma(I)$	θ_{max}(°)	% complete
Global		33829	10192	27.84	66.72%
1	0	4073	2536	27.71	62.74%
2	0	24392	4680	27.84	72.94%
3	0	5364	2976	27.735	59.53%
3	1	2687	1454	27.73	59.67%
3	2	2677	1522	27.735	59.40%

Low completeness statistics are primarily due to the elimination of overlapped reflections in the integration process.

All details can be found in CCDC 1053051-1053052 (stage 1, **1*i*-1*t***), 1053053-1053055 (stage 2, **1*i*-1*t*-1*f***), 1053056-1053057 (stage 3, **1*t*-1*f***) and 1053058-1053059 (**1*f* HS-1*f* LS**) that contain the supplementary crystallographic data for this paper. These data can be obtained free of charge from The Cambridge Crystallographic Data Centre via www.ccdc.cam.ac.uk/data_request/cif.

Refinement process:

The refinements of the Initial and Final structures were done almost identically as previously published, with the exception of how the disordered ClO_4^- were handled. In the present cases, disordered anions were refined isotropically, to reduce the strain on the low data: parameter ratio. A RIGU instruction was also used in both of these cases. The Final data set was truncated at 0.97Å, due to poor I/σ and R_{int}.

The intermediate structure, called **1*t***, is the transition state during a conversion from low spin to high spin. This conversion requires the loss of a solvent molecule (acetone in this case) and creates a sample with severely reduced crystallinity from a nearly perfect crystal. The diffraction of the intermediate was poor beyond 1.2 Å ($I/\sigma < 2.0$, $R_{int} > 30\%$), therefore the data was truncated. This has resulted in very few data points per parameter.

The solvent molecule that is present in the final high spin sample cannot be found in this structure. SQUEEZE[3,4] was used to look for un-modeled electron density, and all that was found was 3 electrons in a void measuring 125.3 $Å^3$ This leads us to conclude that the acetone molecules are so disordered during this meta-stable state, that their scattering contribution is not detected in the Bragg scattering, and these molecules may only contribute diffuse scatter. All hydrogens were assigned geometrically and refined as riding, with U(H) = 1.2*U(C) for aromatic carbons, U(H) = 1.2*U(N) for nitrogens and U(H) = 1.5*U(C) for terminal carbons. A RIGU command was employed in this refinement as well.

The disordered perchlorates were modeled as split sites isotropically, using the following restraints:

SADI_ClO4 0.01 Cl1 O1 Cl1 O2 Cl1 O3 Cl1 O4

SADI_ClO4 0.02 O1 O2 O1 O3 O1 O4 O2 O3 O2 O4 O3 O4

Powder X-ray diffraction.

Temperature-dependent XRPD data from temperature-dependent studies have been applied to monitor the solid-state transformation. A polycrystalline sample of **1*i*** was obtained by fragmenting large single crystals to fill a 0.7 mm borosilicate capillary. XRPD data was collected with an Empyrean PANalytical powder diffractometer, using Cu K_α radiation. An initial dataset was collected at room temperature in the 2θ range 5–40 º to check the phase purity of the sample. Subsequent measurements were performed at 350 K collecting a pattern every 10 minutes. Pawley refinements[5] were performed on each powder pattern using the TOPAS computer program.[6] The observed and calculated diffraction patterns are shown in Figure S15 every 100 min, revealing an excellent fit to a two-phase model, **1*i*** and **1*f***. This indicates the absence of any other detectable crystalline phase thus showing a gradual evolution from **1*i*** to **1*f*** without a trace of the transient phase **1*t***. A detailed view of a selected 2θ range (6.0–10.0º) is presented in Figure 3 in the main text, showing that upon heating at 350 K, the intensity of the peaks corresponding to **1*i*** successively diminishes while new peaks corresponding to **1*f*** grow in intensity.

Differential Scanning Calorimetry (DSC).

DSC experiments were performed with a Q1000 calorimeter from TA Instruments equipped with the LNCS accessory. The temperature and enthalpy scales were calibrated with a standard sample of indium, using its melting transition (156.6 ºC, 3296 J mol^{-1}). The measurements were carried out using aluminum pans either open to the N_2 atmosphere of the setup or closed with a mechanical crimp, with an empty pan as reference.

Optical microscopy and Raman spectroscopy.

Optical images were collected in transmittance mode using the Olympus BH-MA-2 microscope of the Raman spectrometer (see below) and a X5 objective lens. All thermal treatments were done placing the crystals on a quartz plate within a Linkam TS1500V stage. Note that the colours are as captured, but clearly depend on the thickness of the crystal and the exposure time. The exposure time was maintained constant for each crystal so that this parameter was not varied. A few images were also collected in reflectance mode under the same conditions.

Raman spectra were obtained with a DILOR XY spectrometer equipped with a liquid N_2-cooled CCD detector. Scattered light was collected through an X50 microscope objective lens of an Olympus BH-MA-2 microscope. The Si band at 520 cm^{-1} was used for frequency calibration. In a first series of experiments, the 514.53 nm line of an Ar^+ laser was used as the excitation source, operating with the lowest power of the laser, i.e. 1 mW. The corresponding power at the sample surface was estimated in-situ at 0.05 mW, resulting in poor signal. Unfortunately, with larger excitation powers, the material was found to be partially transformed or even partially decomposed, due to the strong absorption of the material in its LS state. Thus, all experiments reported here were done using the 632.8 nm line of a HeNe laser with a 15 mW nominal power as the excitation source. The power at the sample surface was estimated to be ca. 0.45 mW, and was found to have no effect on the sample, being out of the absorption bands of both the LS and HS phases.

Table S1: Crystal data for phases present in two crystals of **1** at different stages of the **1*i*** to **1*f*** transformations

	Crystal 1 stage 1: 1i +1t		Crystal 1 stage 2 : 1i+1t+1f			Crystal 2 stage 3 : 1t+1f	
Crystal size			0.10x0.07x0.05			0.50x0.40x0.30	
	**1*i***	**1*t***	**1*i***	**1*t***	**1*f***	**1*t***	**1*f***
Formula	$C_{36}H_{30}FeN_{10}O_2$,1.35 (C_3H_6O), 2(ClO_4)	2($C_{36}H_{30}FeN_{10}O_2$), 4(ClO_4)	$C_{36}H_{30}FeN_{10}O_2$,1.25 (C_3H_6O), 2(ClO_4)	2($C_{36}H_{30}FeN_{10}O_2$), 4(ClO_4)	$C_{36}H_{30}FeN_{10}O_2$, C_3H_6O, 2(ClO_4)	2($C_{36}H_{30}FeN_{10}O_2$), 4(ClO_4)	$C_{36}H_{30}FeN_{10}O_2$, C_3H_6O, 2(ClO_4)
FW (g mol^{-1})	967.85	1778.90	962.05	1778.90	947.53	1778.90	947.53
T (K)	250	250	250	250	250	293	293
Wavelength (Å)	0.7749	0.7749	0.7749	0.7749	0.7749	0.7749	0.7749
Crystal system	monoclinic	monoclinic	monoclinic	monoclinic	triclinic	monoclinic	triclinic
Space group	$P2_1$/n	$P2_1$/c	$P2_1$/n	$P2_1$/c	*P*–1	$P2_1$/c	*P*–1
a (Å)	14.1613(12)	15.717(3)	14.165(5)	15.679(12)	13.073(3)	15.700(3)	13.0297(8)
b (Å)	21.6112(18)	20.127(3)	21.596(8)	20.102(15)	13.356(3)	20.035(4)	13.2995(9)
c (Å)	15.3855(13)	24.672(4)	15.414(6)	24.601(18)	14.075(3)	24.638(5)	14.0177(9)
α (°)	90	90	90	90	71.88(3)	90	71.801(5)
β (°)	114.3394(11)	90.353(10)	114.290(4)	90.221(8)	69.89(3)	90.45(3)	69.822(4)
γ (°)	90	90	90	90	70.65(3)	90	70.752(4)
V (Å^3)	4290.1(6)	7805(2)	4298(3)	7754(10)	2123.0(10)	7750(3)	2097.8(3)
Z	4	4	4	4	2	4	2
ρ_{calcd} (g cm^{-3})	1.498	1.514	1.487	1.524	1.482	1.525	1.500
μ (mm^{-1})	0.690	0.746	0.688	0.751	0.694	0.752	0.702
Independent reflections	13023 (R_{int}=0.0631)	2904 (R_{int}=0.1751)	2103 (R_{int}=0.0232)	2792 (R_{int}=0.0857)	2413 (R_{int}=0.0419)	2811 (R_{int}=0.0566)	2709 (R_{int}=0.0664)
Data / restraints / parameters	13023 / 403 / 580	2904 / 1482 / 1063	2103 / 904 / 580	2792 / 1482 / 1063	2413 / 1215 / 563	2811 / 1372 / 685	2709 / 714 / 563
Goodness-of-fit on F^2	1.050	1.136	1.229	1.289	1.031	1.051	1.151
Final R_1 / wR_2 [I>2σ(I)]	0.0767 / 0.2215	0.1522 / 0.3469	0.0771 / 0.2095	0.0689 / 0.1596	0.0722 / 0.1877	0.0827 / 0.2019	0.1039 / 0.2907
Final R_1 / wR_2 [all data]	0.0902 / 0.2351	0.1999 / 0.3893	0.1070 / 0.2322	0.1355 / 0.2003	0.1194 / 0.2279	0.1362 / 0.2611	0.1216 / 0.3056
largest diff. peak and hole (*e* Å^3)	1.215 / 1.414	0.469 / –0.452	0.309 / –0.327	0.278 / –0.205	0.390 / –0.388	0.283 / –0.241	0.490 / –0.513

Table S2: Crystal data for compound **1*f*** in its HS and LS states, respectively at 260 and 150 K.

	**1*f* HS**	**1*f* LS**
Crystal size	0.18x0.14x0.08	
Formula	$C_{36}H_{30}FeN_{10}O_2$, C_3H_6O, $2(ClO_4)$	
FW (g mol^{-1})	947.53	
T (K)	260	150
Wavelength (Å)	0.7749	
Crystal system	triclinic	
Space group	P–1	
a (Å)	13.085(6)	12.906(9)
b (Å)	13.381(6)	13.199(9)
c (Å)	14.139(7)	14.759(10)
α (°)	72.092(6)	71.402(8)
β (°)	69.882(7)	68.242(8)
γ (°)	70.219(6)	67.214(8)
V (Å^3)	2135.6(17)	2107(3)
Z	2	
ρ_{calcd} (g cm^{-3})	1.473	1.493
μ (mm^{-1})	0.690	0.699
Independent reflections	5051 (R_{int}=0.0779)	5640 (R_{int}=0.0788)
Data / restraints / parameters	5051 / 220 / 603	5640 / 0 / 584
Goodness-of-fit on F^2	1.077	1.033
Final R_1 / wR_2 [I>2σ(I)]	0.0911 / 0.2534	0.0819 / 0.2314
Final R_1 / wR_2 [all data]	0.1404 / 0.2849	0.1252 / 0.2632
largest diff. peak and hole (e Å^3)	0.773 / –0.448	0.729 / –0.424

Table S3. Selected bond distances and angles in the structures of **1*i***, **1*t*** and **1*f*** at the intermediate stage (**1*i***+**1*t***+**1*f***) of the **1*i*** to **1*f*** transformation.

	**1*i*** (Fe1)	**1*f*** (Fe1)	**1*t*** (Fe01 site)	**1*t*** (Fe02 site)
Fe–N3 (or N103)	1.923(12)	2.108(11)	1.950(17)	2.120(19)
Fe–N8 (or N108)	1.940(12)	2.123(12)	1.946(18)	2.135(18)
Fe–N4 (or N104)	1.961(10)	2.217(11)	1.97(2)	2.221(19)
Fe–N7 (or N107)	1.964(10)	2.145(11)	1.963(17)	2.124(18)
Fe–N2 (or N102)	1.997(10)	2.198(11)	1.946(19)	2.188(19)
Fe–N9 (or N109)	2.004(10)	2.169(9)	1.961(18)	2.159(17)
N3–Fe–N8	176.9(4)	171.9(3)	173.7(8)	173.8(8)
N3–Fe–N4	79.6(4)	73.7(4)	79.1(8)	74.2(8)
N8–Fe–N4	99.3(5)	102.1(4)	95.6(8)	111.9(7)
N3–Fe–N7	98.6(4)	113.2(4)	103.5(7)	104.4(7)
N8–Fe–N7	78.5(5)	73.9(4)	80.2(8)	74.2(7)
N4–Fe–N7	89.6(4)	98.2(4)	94.9(7)	93.5(7)
N3–Fe–N2	79.5(4)	74.6(4)	79.5(9)	72.7(8)
N8–Fe–N2	101.6(4)	109.6(4)	105.9(8)	101.3(7)
N4–Fe–N2	159.1(5)	148.2(4)	158.5(8)	146.8(8)
N7–Fe–N2	94.1(4)	92.7(4)	88.4(7)	97.3(6)
N3–Fe–N9	103.8(5)	99.1(4)	98.2(8)	106.5(7)
N8–Fe–N9	79.1(5)	73.7(4)	78.4(8)	74.6(7)
N4–Fe–N9	93.5(4)	89.0(4)	90.3(7)	99.5(7)
N7–Fe–N9	157.6(5)	147.7(5)	158.3(8)	148.7(8)
N2–Fe–N9	90.9(4)	92.7(4)	94.4(7)	87.2(6)

Table S4. Selected bond distances and angles in the structures of **1*f* LS** (150 K) and **1*f* HS** (260 K).

	**1*f* LS** (150 K)	**1*f* HS** (260 K)
Fe1–N8	1.909(6)	2.147(8)
Fe1–N3	1.940(6)	2.107(7)
Fe1–N7	1.955(6)	2.141(8)
Fe1–N4	1.956(6)	2.216(7)
Fe1–N9	1.972(6)	2.146(7)
Fe1–N2	2.000(6)	2.203(8)
N8–Fe1–N3	176.0(3)	172.0(3)
N8–Fe1–N7	79.1(3)	74.0(3)
N3–Fe1–N7	101.9(3)	112.6(3)
N8–Fe1–N4	97.4(3)	101.3(3)
N3–Fe1–N4	78.7(3)	73.8(3)
N7–Fe1–N4	93.4(3)	97.8(3)
N8–Fe1–N9	79.6(3)	74.5(3)
N3–Fe1–N9	99.4(3)	98.8(3)
N7–Fe1–N9	158.7(3)	148.5(3)
N4–Fe1–N9	90.7(2)	89.2(3)
N8–Fe1–N2	103.9(3)	109.5(3)
N3–Fe1–N2	79.9(3)	75.5(3)
N7–Fe1–N2	90.6(2)	92.1(3)
N4–Fe1–N2	158.7(3)	149.2(3)
N9–Fe1–N2	93.2(2)	97.3(3)

Table S5. Hydrogen bonding in the structures of **1*i***, **1*t*** and **1*f*** at the intermediate stage (**1*i***+**1*t***+**1*f***) of the **1*i*** to **1*f*** transformation.

D–H···A	D–H (Å)	H···A (Å)	D–A (Å)	D–H···A (º)
**1*i***				
N1–H1···O4_3	0.87	2.06	2.91(2)	165
N5–H5···O2	0.87	2.03	2.603(18)	123
N6–H6···O1_1	0.87	1.92	2.78(3)	168
N10H10···O3	0.87	1.92	2.75(2)	159
**1*f***				
N1–H1D···O1_2	0.87	2.02	2.83(3)	154
N5–H5B···O2	0.87	2.00	2.587(16)	124
N6–H6B···O2_1	0.87	2.58	3.36(3)	149
N6–H6B···O4_1	0.87	2.10	2.91(2)	154
N10–H10A···O3	0.87	1.88	2.749(17)	177
**1*t***				
N1–H1B···O1	0.87	2.17	2.64(3)	113
N1–H1B···O1_1	0.87	2.26	3.08(2)	158
N5–H5A···O2	0.87	1.96	2.59(3)	128
N6–H6A···O1_4	0.87	1.95	2.80(2)	164
N6–H6A···O2_4	0.87	2.36	2.94(2)	124
N10–H10C···O1_3	0.87	1.96	2.83(2)	172
N101–H10D···O101	0.87	2.02	2.55(3)	118
N105–H10E···O4_4	0.87	2.50	3.29(3)	152
N105–H10E···O102	0.87	2.17	2.67(3)	116
N106–H10F···O4_1	0.87	2.05	2.87(2)	156
N110–H11A···O1_2	0.87	2.44	3.03(2)	125
N110–H11A···O3_2	0.87	2.03	2.89(2)	170

Table S6. Hydrogen bonding in the structures of **1*f* LS** (150 K) and **1*f* HS** (260 K).

D–H···A	D–H (Å)	H···A (Å)	D–A (Å)	D–H···A (º)
**1f LS (150 K)**				
N1–H1A···O8	0.91(10)	2.06(9)	2.939(11)	162(7)
N5–H5B···O2	0.97(10)	2.12(8)	2.579(8)	107(6)
N6–H6B···O5	0.93(9)	2.04(9)	2.909(12)	155(9)
N6–H6B···O6	0.93(9)	2.50(10)	3.281(14)	141(7)
N10–H10A···O1S#1	1.07(9)	1.68(9)	2.714(11	160(7)
**1f HS (260 K)**				
N1–H1A···O8	0.97(11)	2.17(10)	2.928(14)	134(8)
N5–H5B···O2	0.95(10)	1.97(9)	2.605(10)	122(8)
N6–H6B···O6B	1.08(13)	2.23(13)	3.24(2)	155(10)
N10–H10A···O1S#1	1.00(11)	1.79(11)	2.773(14)	166(9)

#1: 1+x,y,-1+z

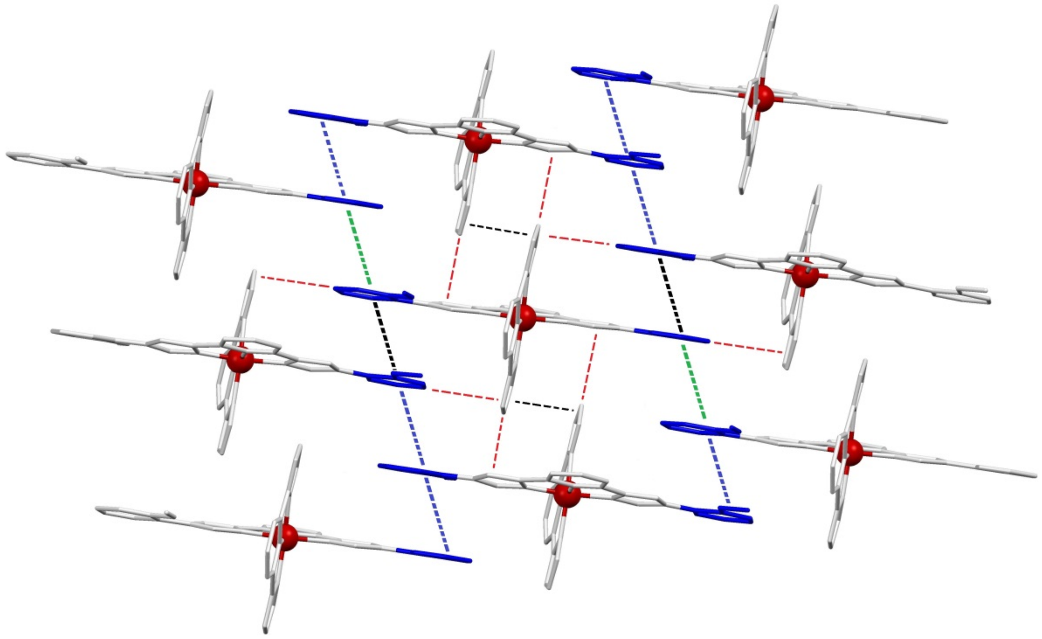

Figure S1. Molecular representation of the $[Fe(\textit{bpp})(H_2L)]^{2+}$ cation in **1*i*** as disposed within the supramolecular sheets present in the lattice, emphasizing the four π···π interactions (black dashed lines) and eight C–H···π interactions (red dashed lines) formed by each cation (here represented by the central one) with its four first neighbors and the two π···π interactions (green dashed lines) with its two second neighbours. Also represented are the stripes of methoxophenyl groups (blue) running throughout the sheets, connected by a series of π···π contacts (thick dashed lines), which include some of these previously mentioned.

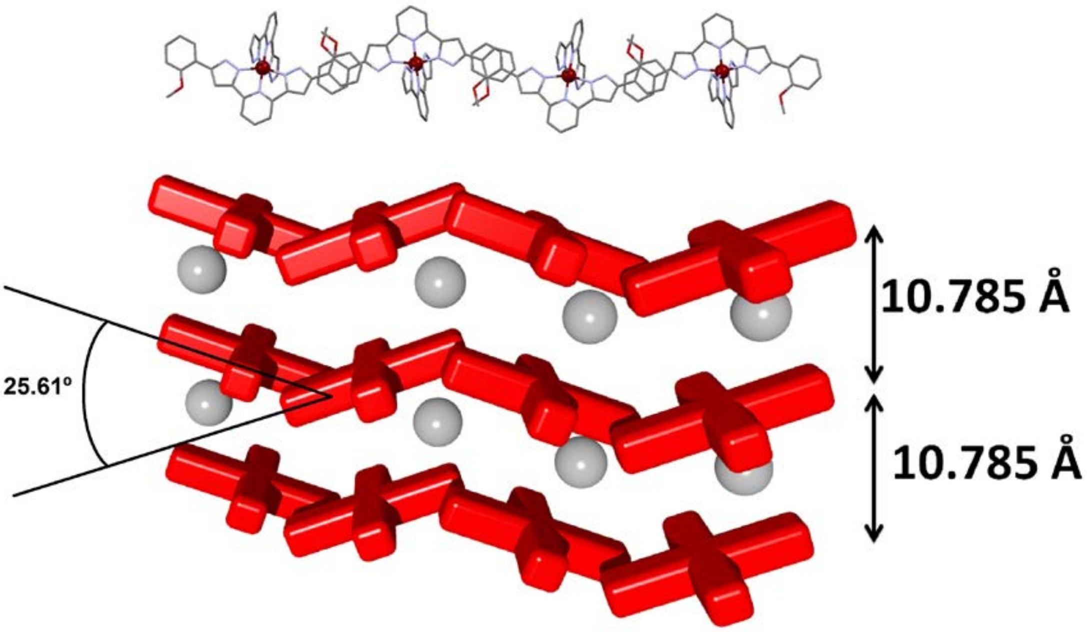

Figure S2. (top) Representation of a linear array of $[Fe(\textit{bpp})(H_2L)]^{2+}$ cations present within sheets of **1*i***, emphasizing the two different orientations featured by these complexes within the lattice. (bottom) Schematic representation of three lines of cross-shaped $[Fe(\textit{bpp})(H_2L)]^{2+}$ cations within three corrugated parallel sheets, showing the distance between them and the angle formed by differently oriented complexes (see text). Loosely bound and disordered molecules of acetone in between sheets are also represented.

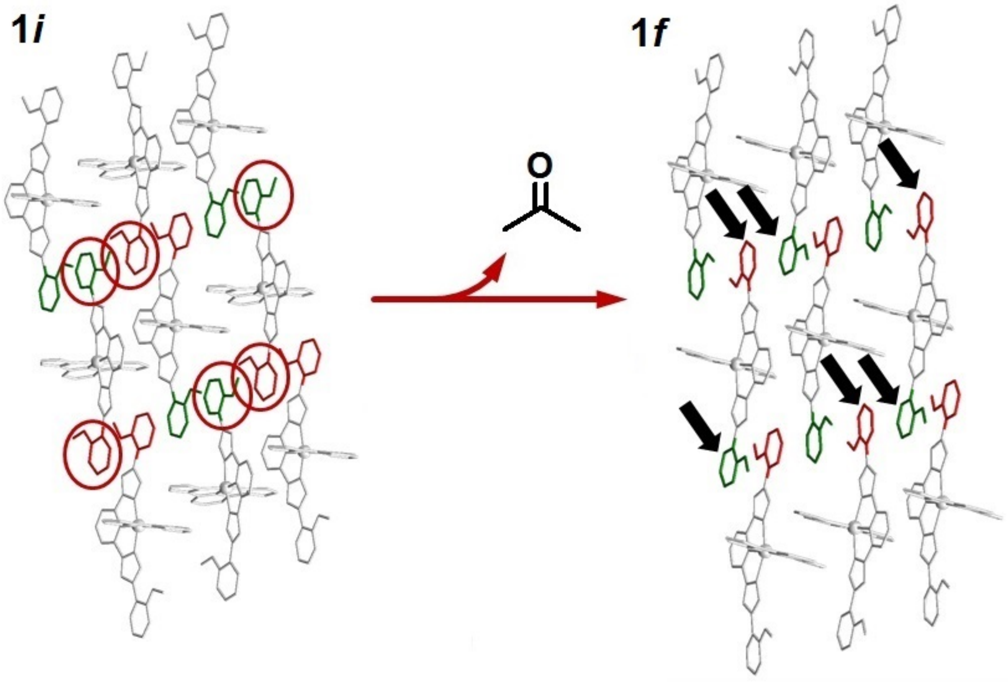

Figure S3. Representation of $[Fe(\textit{bpp})(H_2L)]^{2+}$ cations in **1*i*** and **1*f***, emphasizing the rotation by 180º of 50% of the distal methoxophenyl groups of H_2L in passing from one to the other. Green and red represent the two orientations of the methoxophenyl groups with respect to the plane of the sheet.

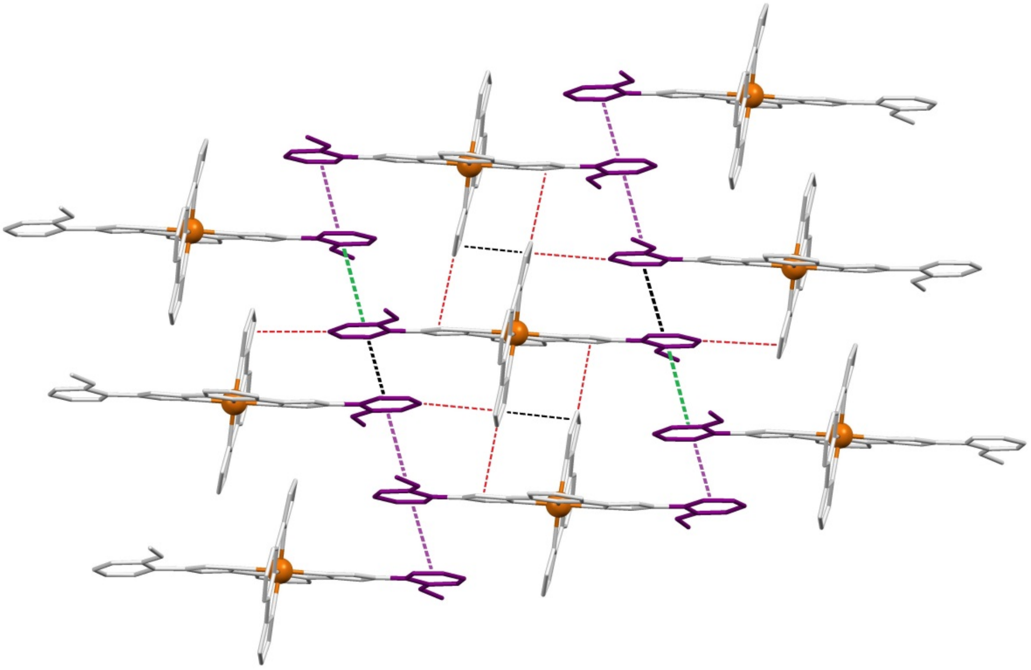

Figure S4. Molecular representation of the [Fe(*bpp*)(H_2L)]$^{2+}$ cation in **1*f*** within the sheets of the lattice. The intermolecular interactions with neighboring cations are shown as in Fig S1.

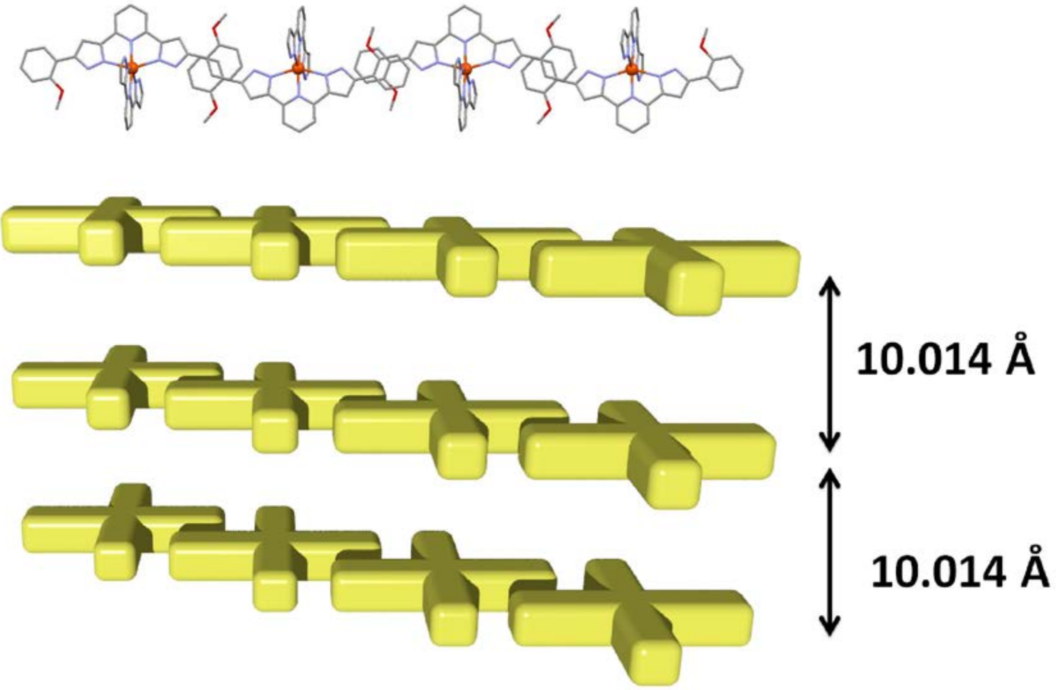

Figure S5. (top) Representation of a linear array of [Fe(*bpp*)(H_2L)]$^{2+}$ parallel cations present within sheets of **1*f***. (bottom) Schematic representation of three lines of cross-shaped [Fe(*bpp*)(H_2L)]$^{2+}$ cations within three parallel sheets, illustrating the change in orientation experienced upon the **1*i*** → **1*f*** transformation.

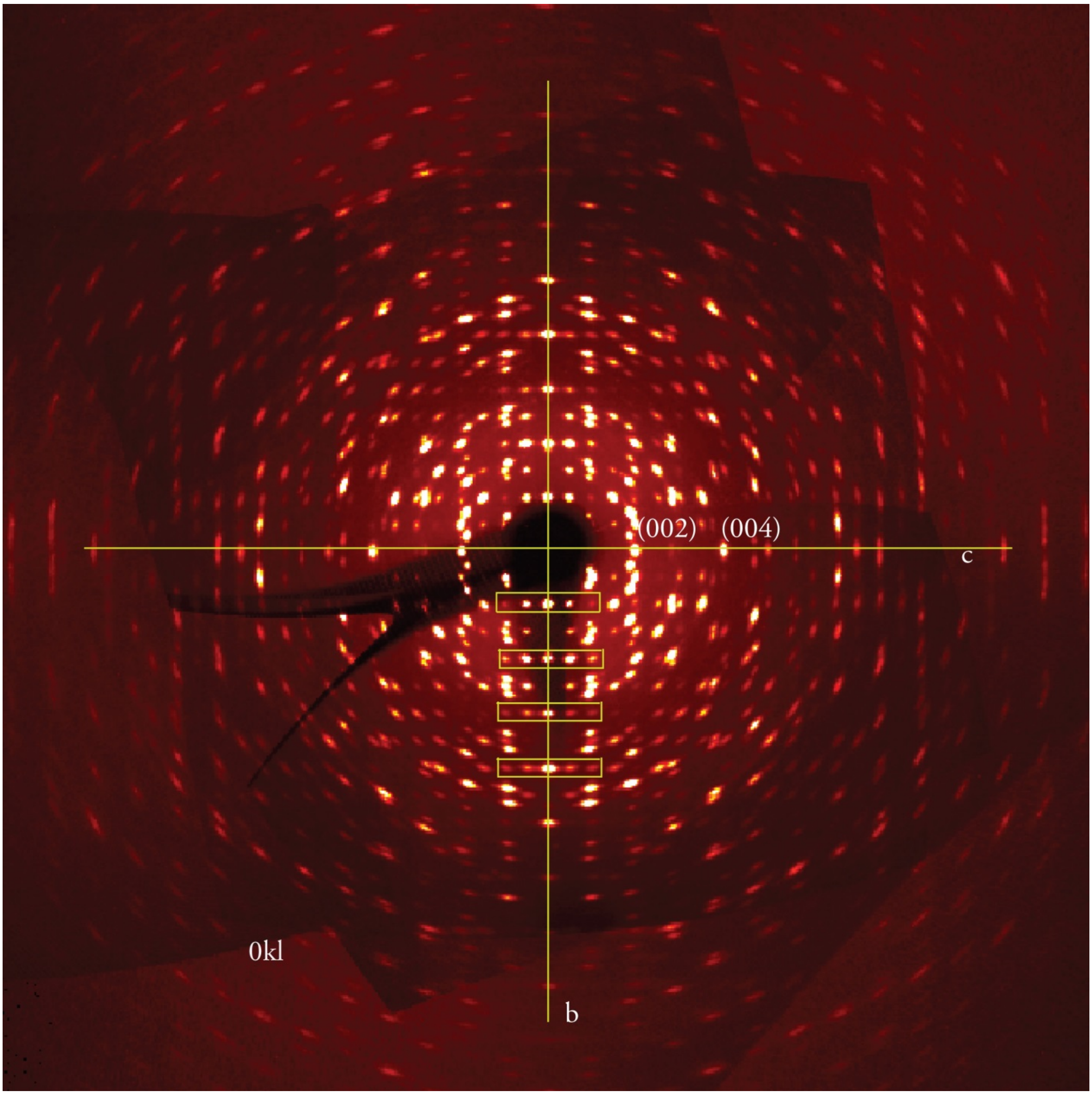

Figure S6. Reciprocal space pseudo-precession view of the *0kl* layer of phase **1*t***, where the twinned components of phase **1*f*** also appear. The systematic absences of $P2_1/c$ are shown along the *b* and *c* axes. There are reflections around the 02*n*0 reflections that might correspond to satellites due to some level of modulation in the structure. This could be a possible explanation for the lack of detection of the acetone molecules. Many of these apparent satellites are however likely arising from the twinned components of phase **1*f*** present at different depths in reciprocal space.

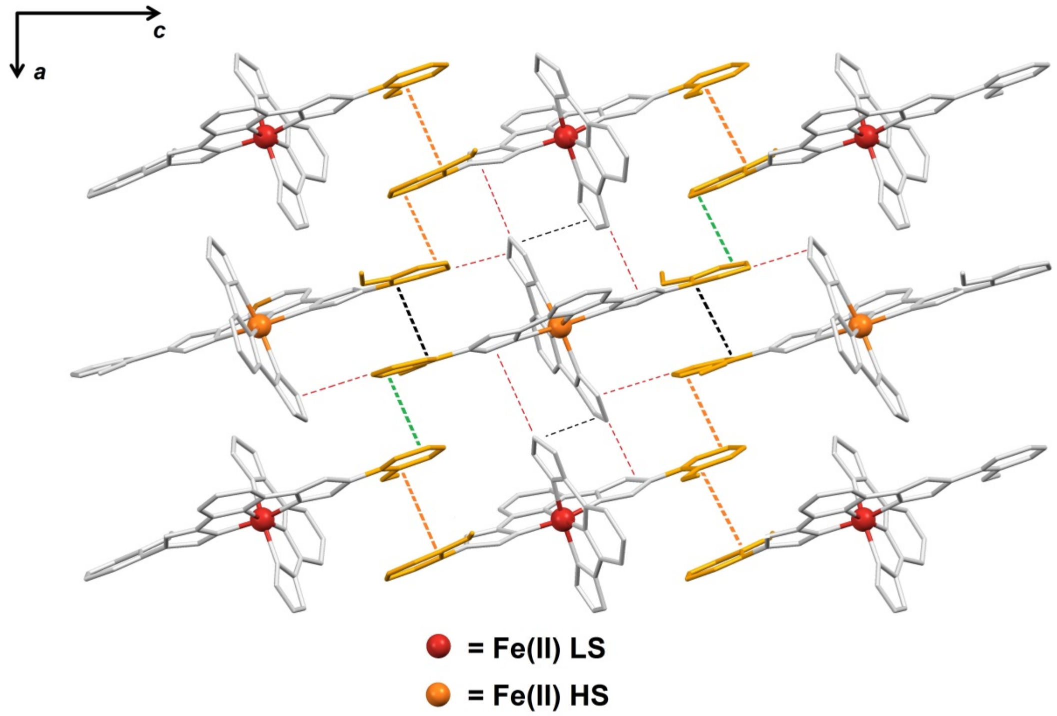

Figure S7. Molecular representation of the [Fe(*bpp*)(H_2L)]$^{2+}$ cation in **1*t*** within the sheets of the lattice. The intermolecular interactions with neighboring cations are shown as in Fig S1. The ordered distribution of HS and LS centers is emphasized.

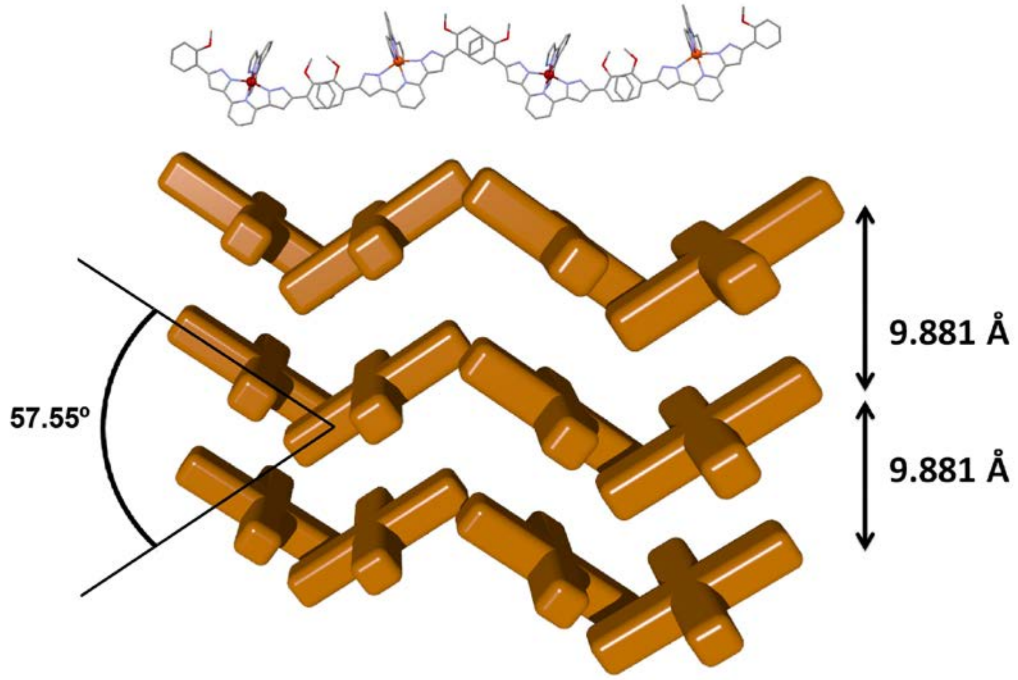

Figure S8. (top) Representation of a linear array of [Fe(*bpp*)(H_2L)]$^{2+}$ cations present within sheets of **1*t***, emphasizing various different orientations (largest angle between cations shown), two spin states (HS, orange; LS, red) and high corrugation. (bottom) Schematic representation of three lines of cross-shaped [Fe(*bpp*)(H_2L)]$^{2+}$ cations within three parallel sheets, illustrating the enhanced corrugation and diminished distance between sheets.

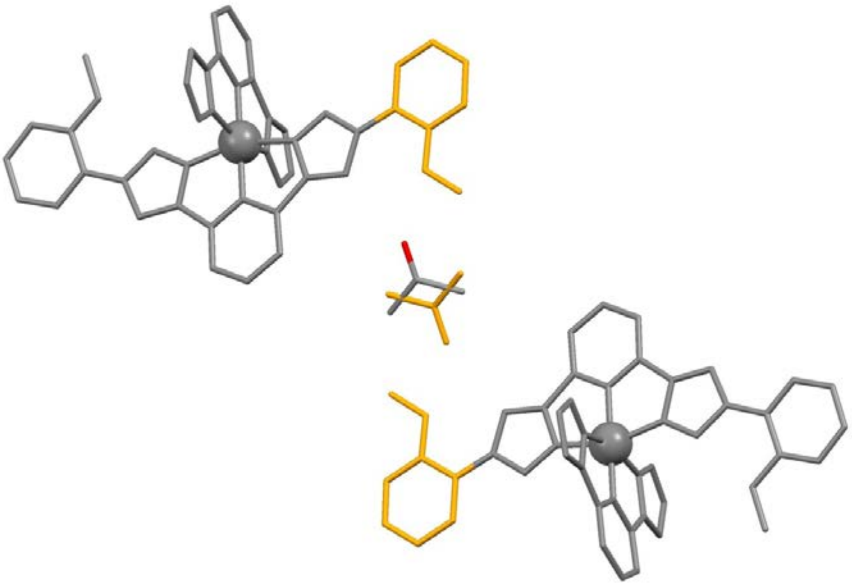

Figure S9. Molecular representation of two [Fe(*bpp*)(H_2L)]$^{2+}$ cations in **1*i*** from two adjacent sheets of the lattice, and their closest disordered acetone species. The yellow methoxyphenyl groups rotate 180º during the **1*i*** → **1*t*** transformation.

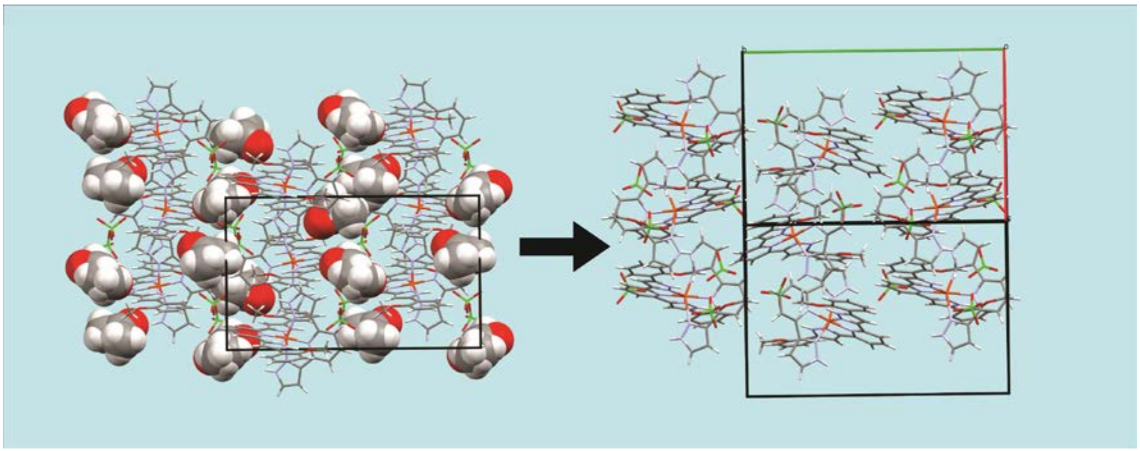

Figure S10. Representation of the **1*i*** → **1*t*** transformation showing the unit cells of the two phases involved, oriented along the same viewing perspective in order to emphasize the relationship between their vectors. Thus the reciprocal lattice vectors 010 and $11\bar{1}$ of **1*i*** were found to be nearly parallel to 010 and $0\bar{1}\bar{2}$, respectively, of **1*t*.** The acetone molecules are shown in space-filling mode, for clarity.

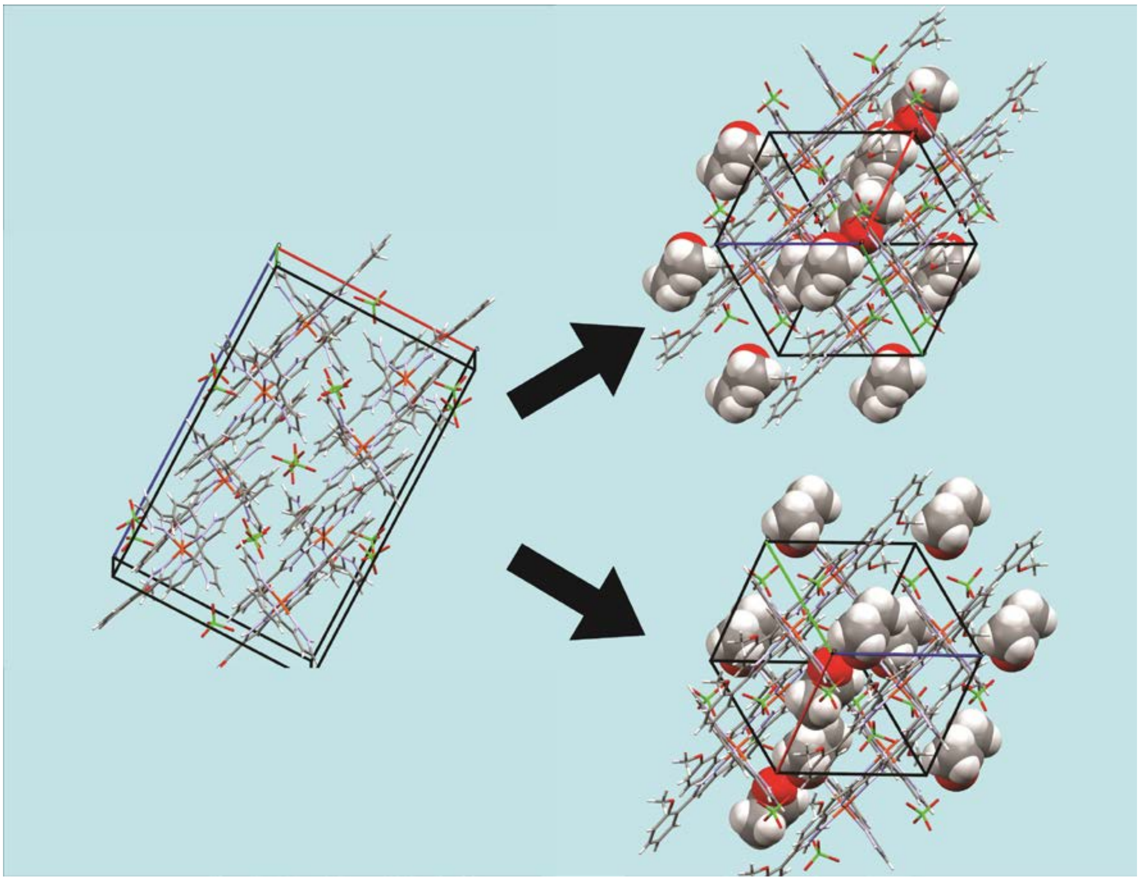

Figure S11. Representation of the **1*t*** → **1*f*** transformation showing the unit cells of the two phases involved, oriented along the same viewing perspective in order to emphasize the relationship between their vectors. Here, the final phase **1*f*** is in fact a twin, with both domains related by the lost 2-fold rotation. The latter is coincident with the 111 reciprocal axis for both domains. The reciprocal lattice vector 020 in **1*t*** is nearly parallel with $\overline{111}$ of both domains, while $1\bar{2}3$ of **1*t*** is nearly parallel to 021 of the first domain and 201 of the second domain of **1*t***. The acetone molecules are shown in space-filling mode, for clarity.

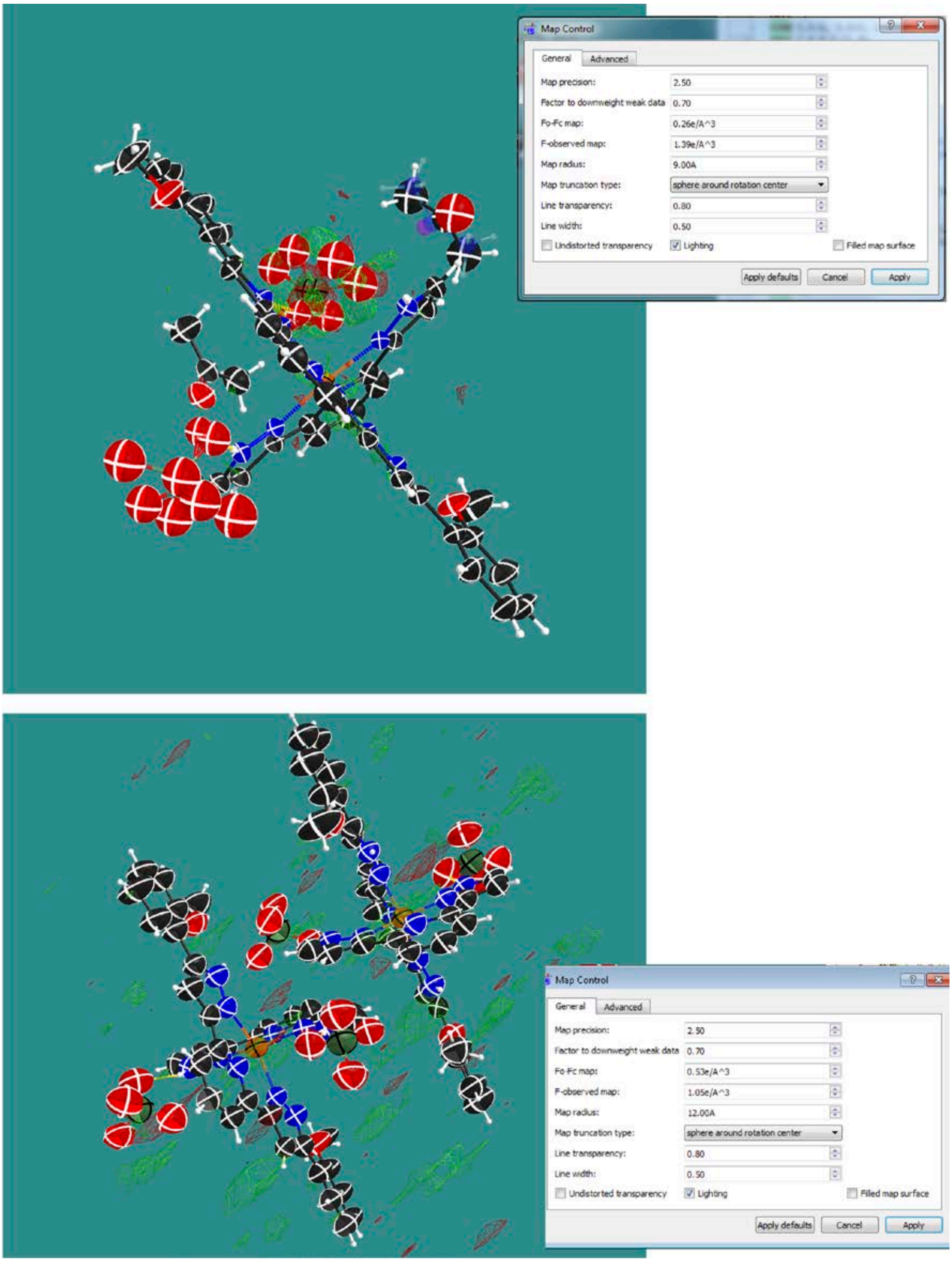

Figure S12. Fo – Fc maps viewed along the *b* axis for the structures of the initial phase **1*i*** (top) and intermediate phase **1*t*** (bottom) at stage 1 of the transformation.

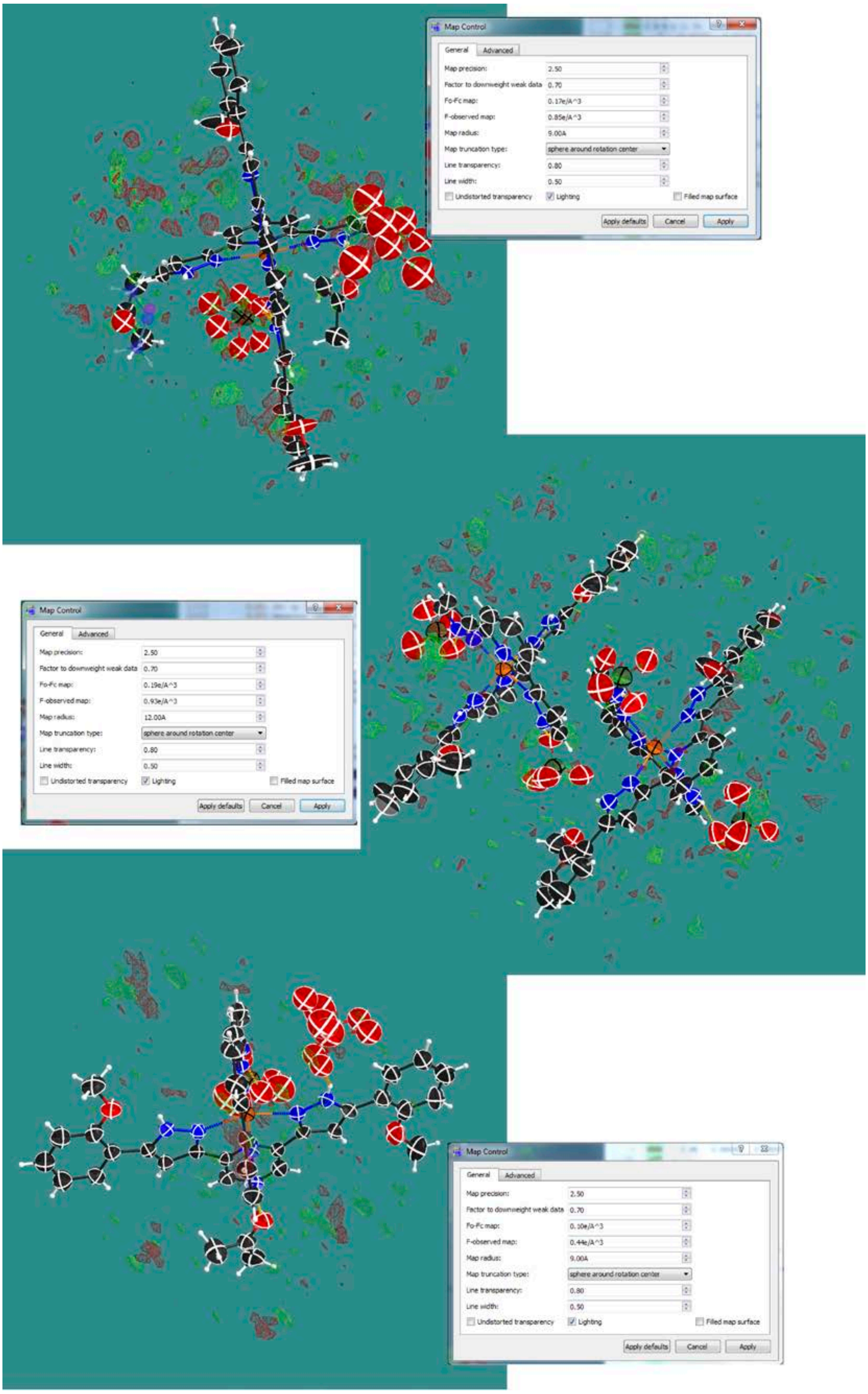

Figure S13. Fo – Fc maps viewed along *b* axis for the structures of the initial phase **1*i*** (top), intermediate phase **1*t*** (middle) and final phase **1*f*** (bottom) at stage 2 of the transformation.

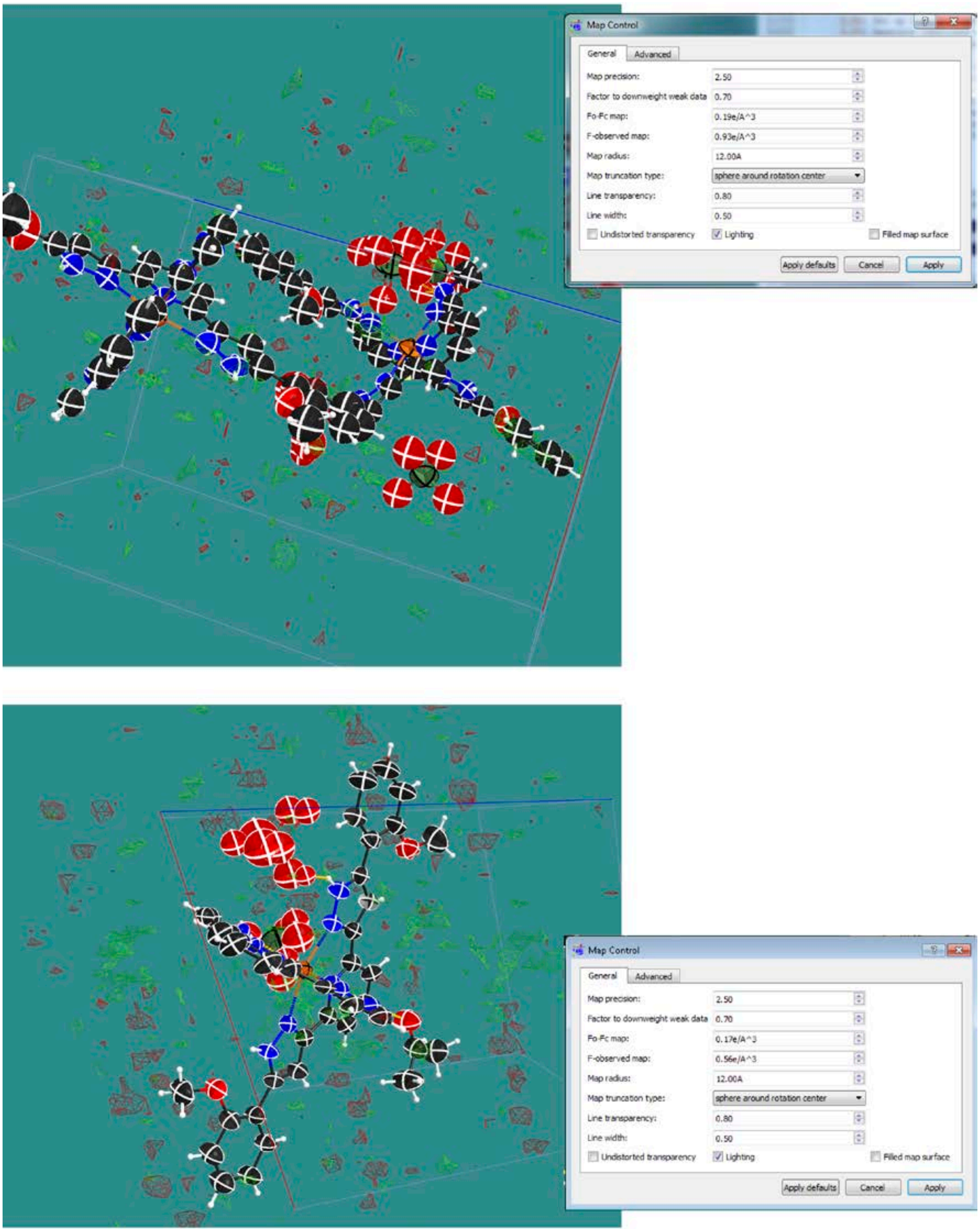

Figure S14. Fo – Fc maps viewed along *b* axis for the structures of the intermediate phase **1*t*** (top) and final phase **1*f*** (bottom) at stage 3 of the transformation.

1*i* 298 K

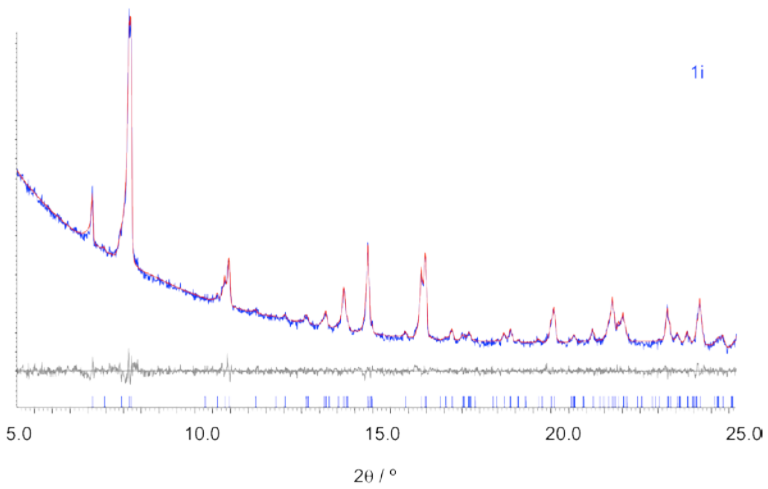

1*i* 353 K

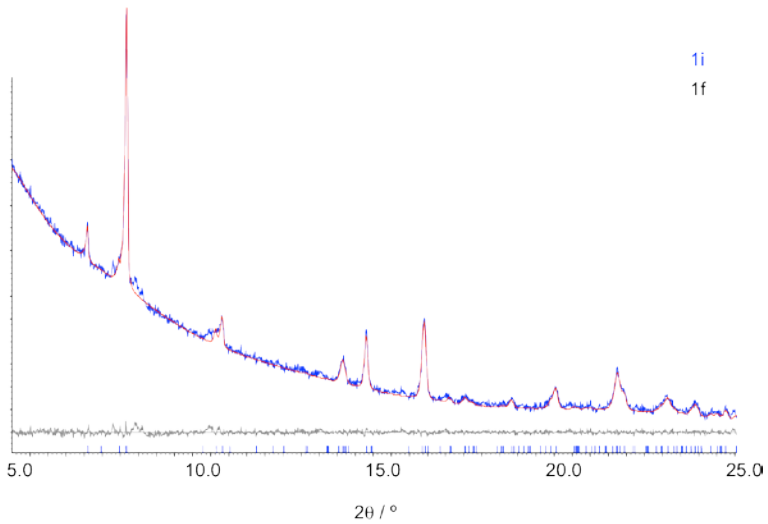

100 min

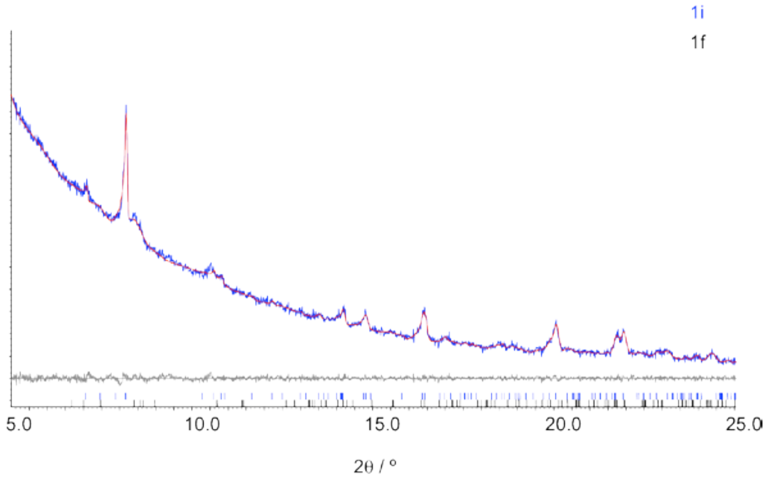

200 min

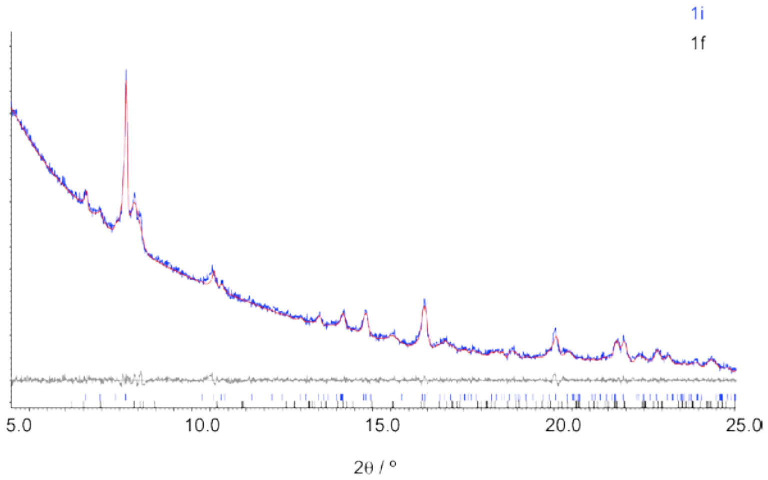

300 min

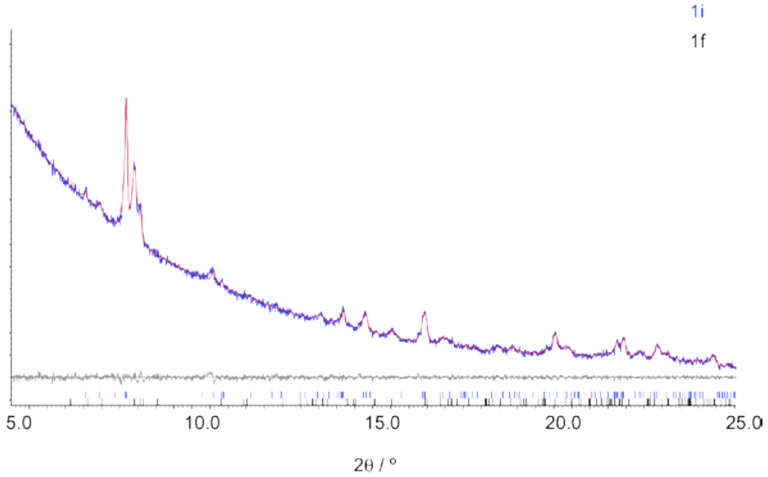

400 min

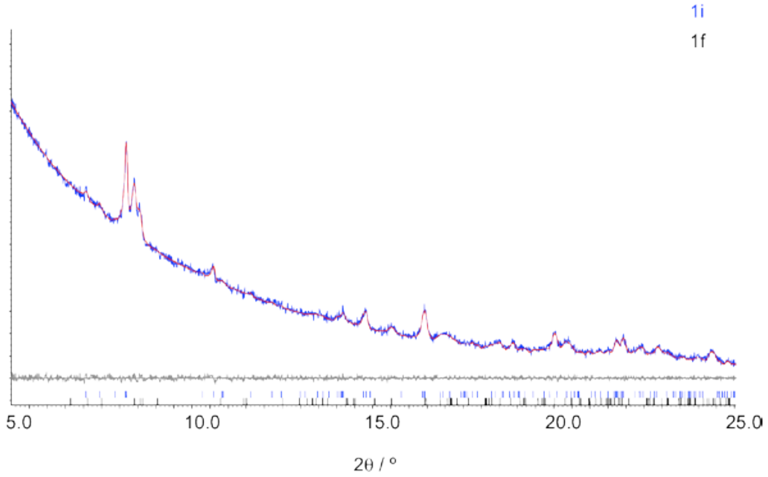

500 min

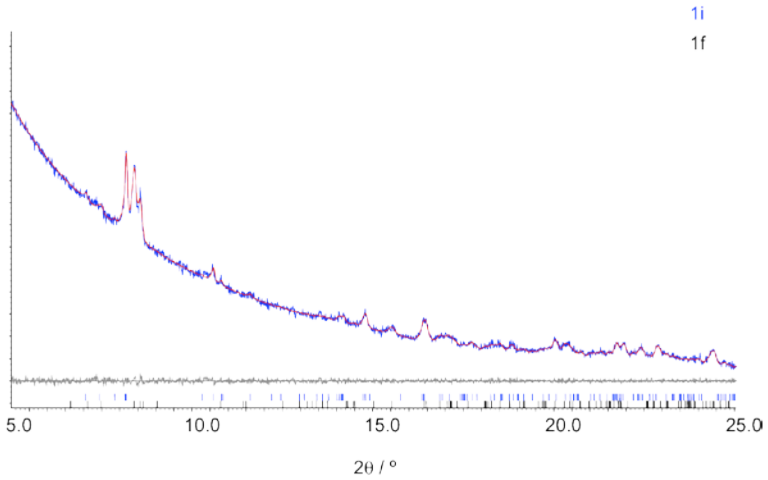

600 min

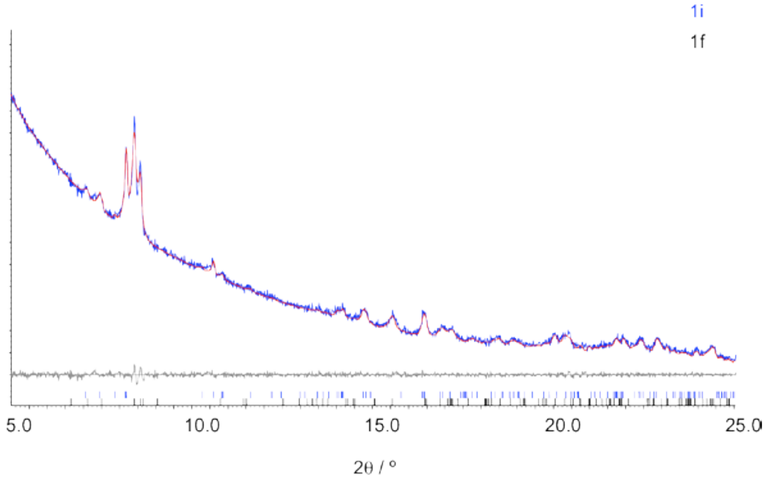

700 min

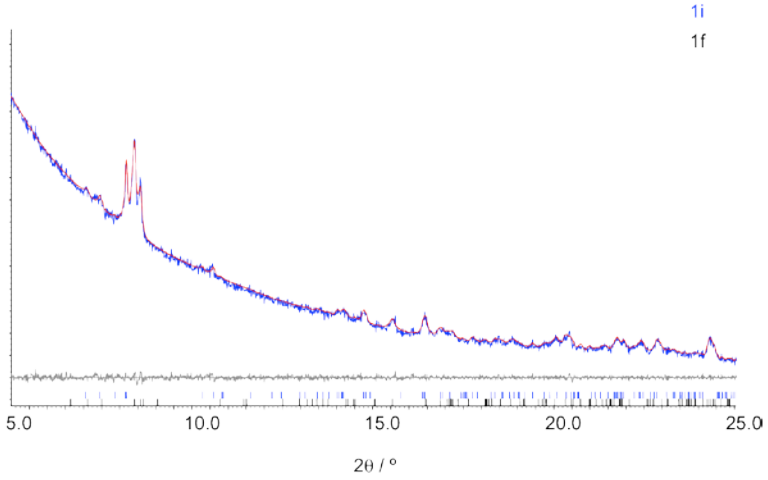

800 min

900 min

1000 min

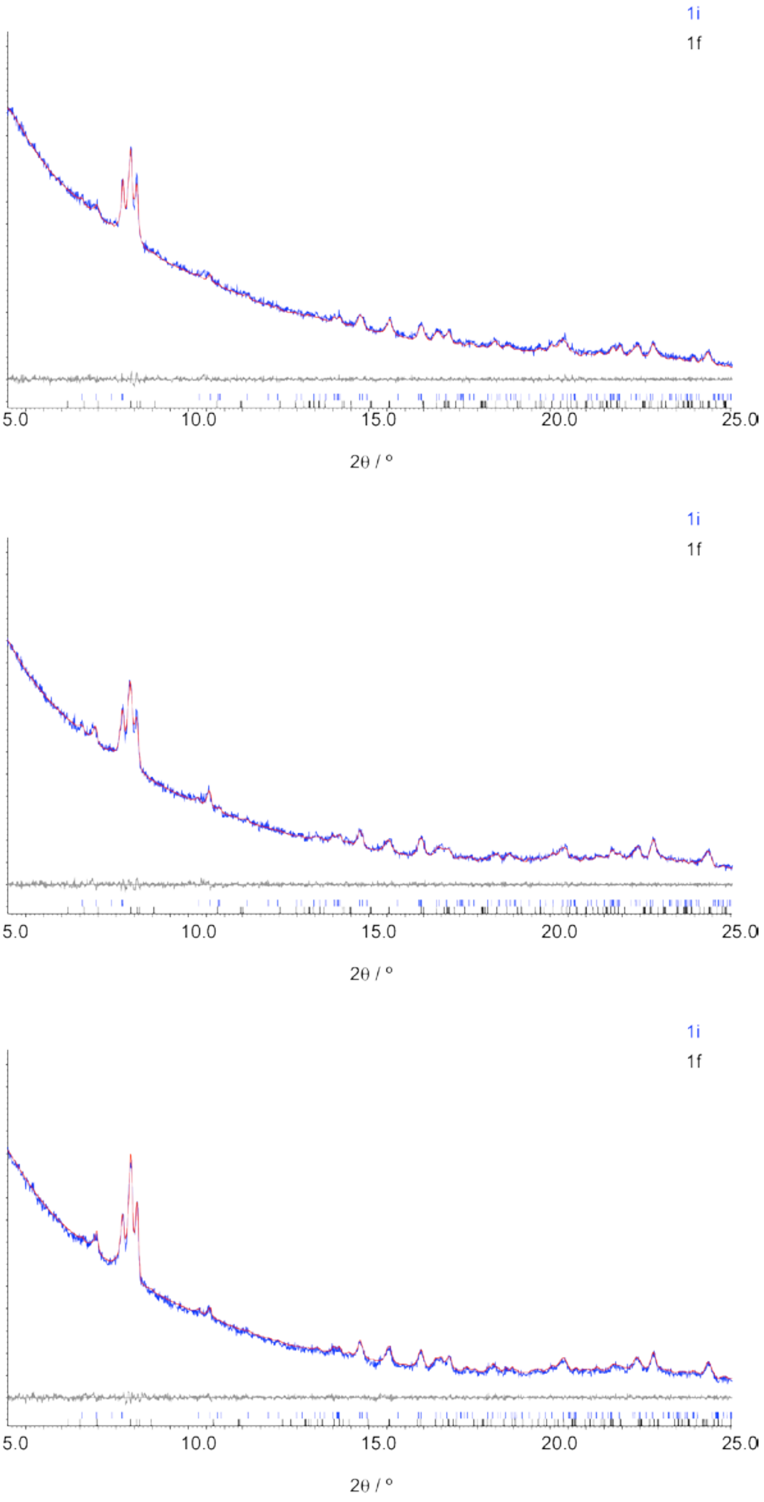

Figure S15. Observed (blue) and calculated (red) profiles and difference plots (I_{obs} − I_{calcd}; gray) of the Pawley refinements (2θ range 5.0−25.0°) for the reaction **1*i*** → **1*f*** at different reaction times.

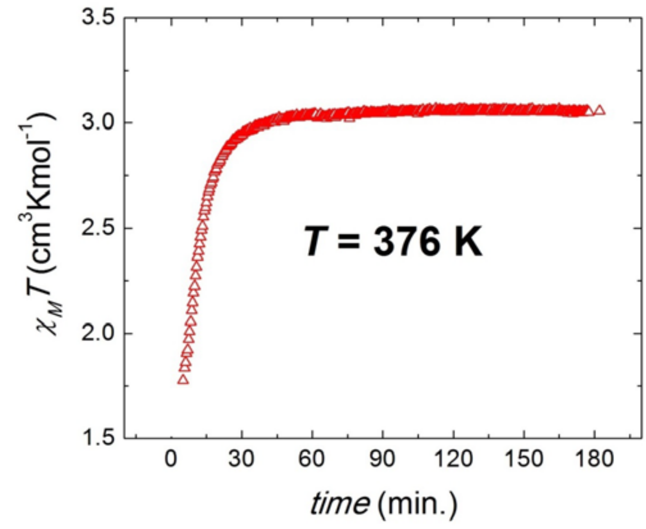

Figure S16. Variation of χT with time at the constant temperature T = 376 K after reaching this value gradually at 1 K/min.

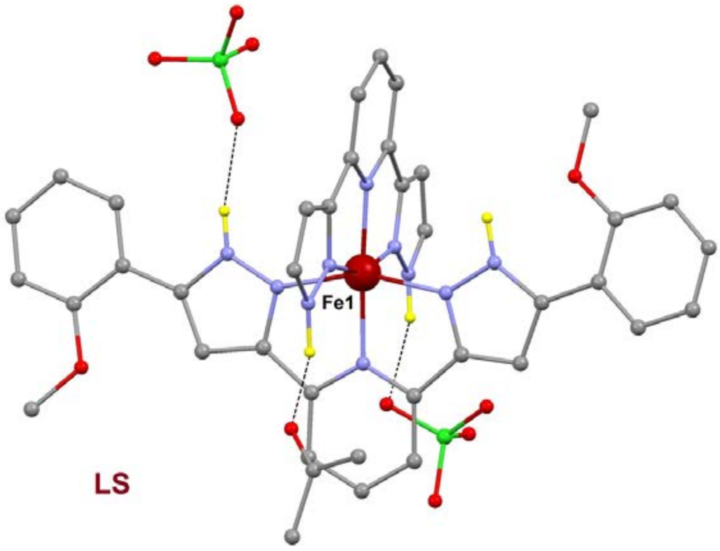

Figure S17. Representation of the unit cell of [Fe(*bpp*)(H_2L)](ClO_4)$_2$·C_2H_6O (1*f*) at 100K. The Fe(II) center is now in the LS. Grey, C; red, O; green, Cl; purple, N; red Fe(II), LS. Only hydrogen atoms on N–H groups shown in yellow. Hydrogen bonds shown with dashed lines.

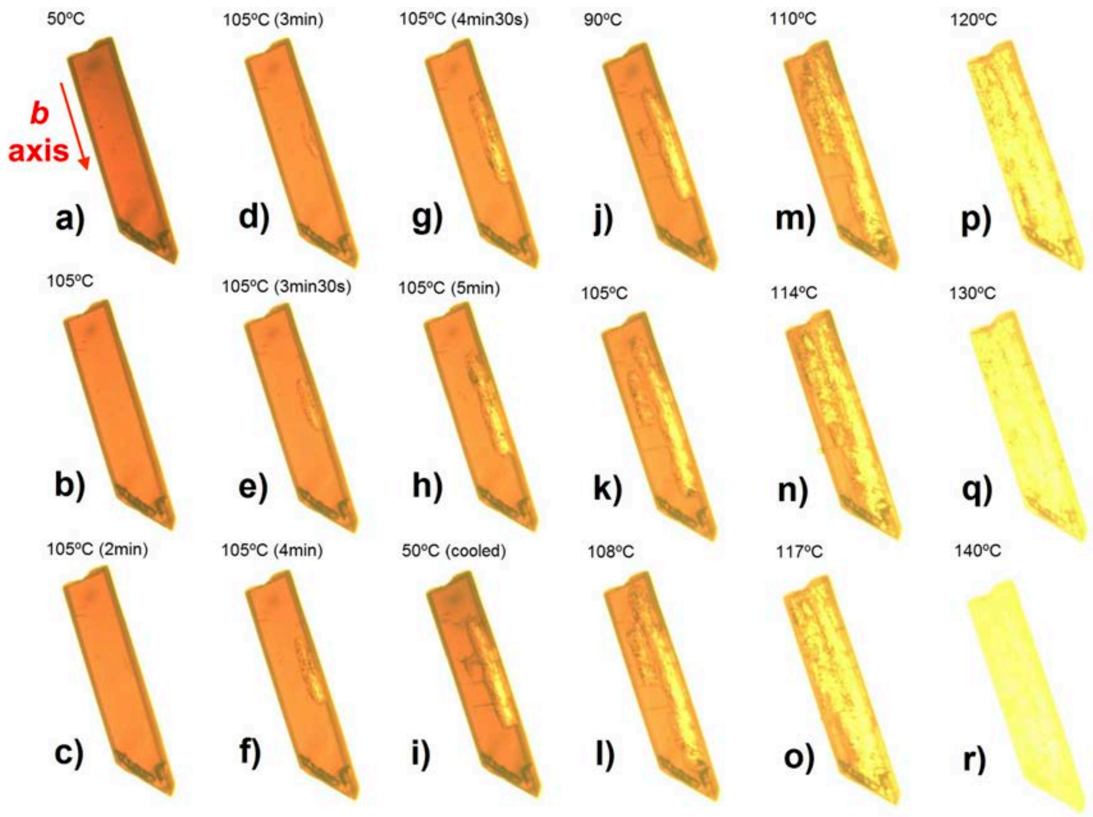

Figure S18. Transmission optical microscopy images taken on a single crystal of **1*i*** covered with little Paraton N grease at 50ºC a), then passed different amounts of time at 105ºC b) through h), after being cooled back to 50ºC i), and finally at several temperatures during the subsequent warming process up to 140ºC j) through r). These images capture different moments of the SCSC sequential transformation corresponding to **1*i*** → (**1*i*** + **1*t***) → (**1*i*** + **1*t*** + **1*f***) → (**1*t*** + **1*f***) → **1*f***.

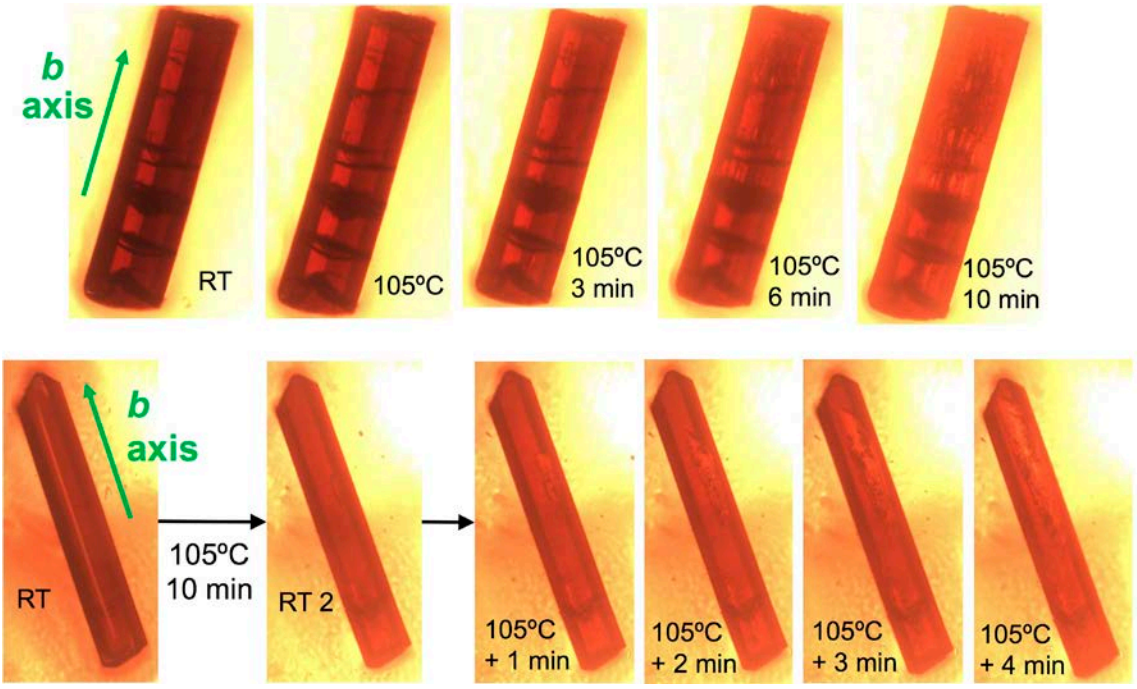

Figure S19. Transmission optical microscopy images taken on two single crystals of **1*i*** covered with little Paraton N grease and having been submitted to a common thermal treatment: warming to 105ºC, isotherm at 105ºC for 10 min, cooling to RT, warming again to 105ºC, isotherm at 105ºC for 4 min. Not shown here are a further cooling to RT and finally warming to 140ºC at 5ºCmin^{-1}. Top: the crystal features many cracks and fissures causing the **1*i*** → **1*f*** transformation to be almost complete after the first warming step. Bottom: the crystal is of great quality, which slows down significantly the transition; after the first warming step only a small portion of the crystal has transformed (RT 2) and a second warming step is necessary to bring the transformation further significantly.

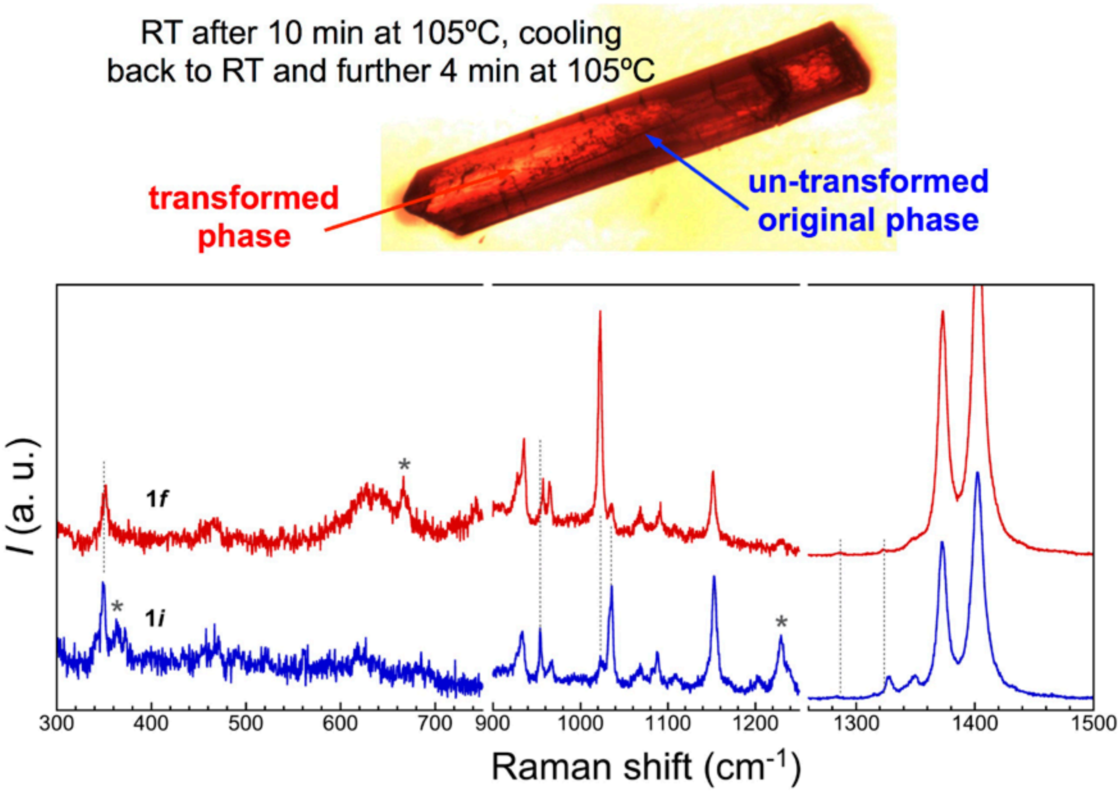

Figure S20. Top: transmission optical microscopy image of a single crystal of **1*i*** covered with little Paraton N grease and after the indicated thermal treatment (same crystal as in Fig. S12 bottom). The indicated areas have Raman spectra characteristic of either the initial phase **1*i*** or the transformed phase **1*f***. Bottom: Characteristic Raman spectra of the un-transformed initial **1*i*** and transformed **1*f*** phases.

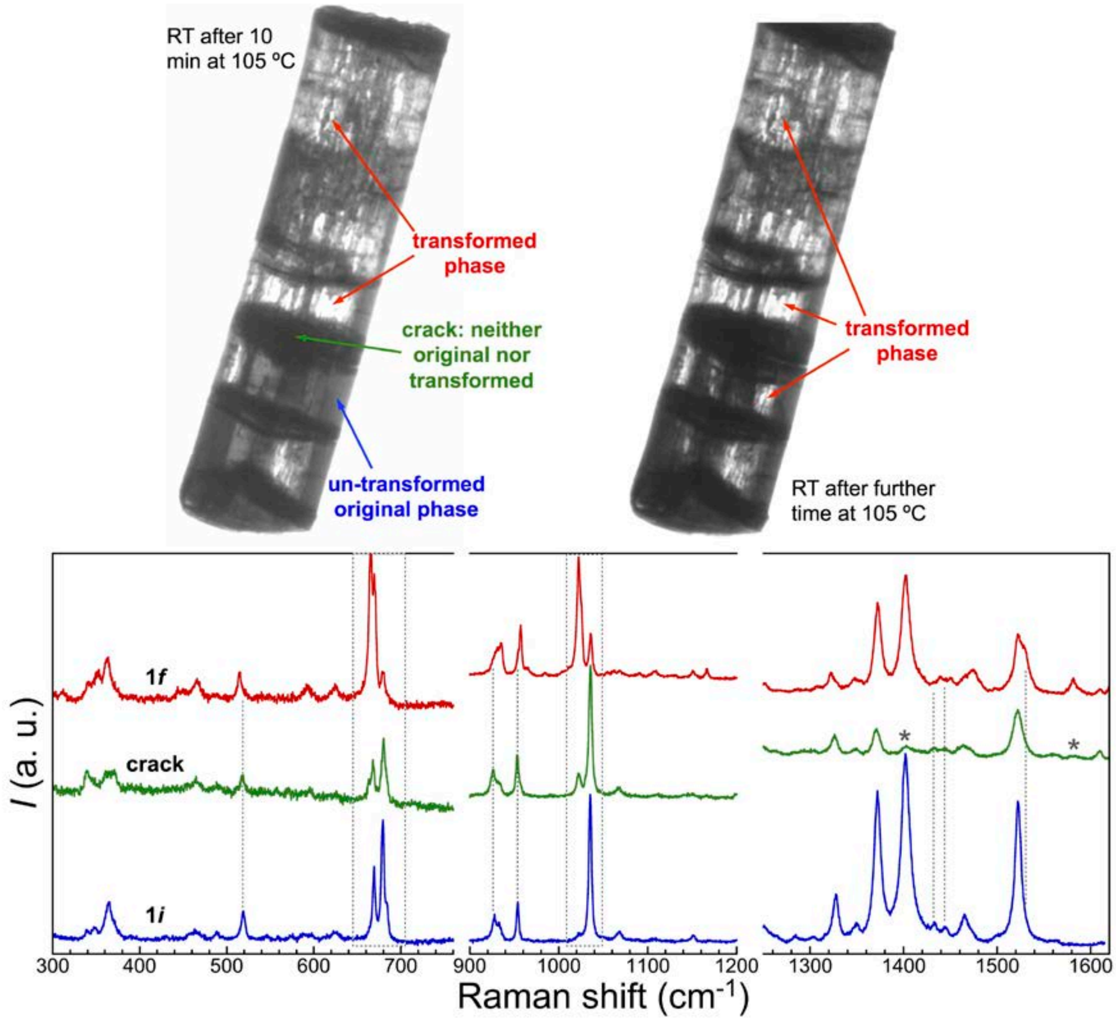

Figure S21. Top: transmission optical microscopy images of a single crystal of **1*i*** covered with little Paraton N grease and after the indicated thermal treatment (same crystal as in Fig. S12 top). The indicated areas have Raman spectra characteristic of either the initial phase **1*i*** or the transformed phase **1*f***. Bottom: Characteristic Raman spectra of the crack areas compared with those of the initial **1*i*** and transformed **1*f*** phases. Dashed vertical lines and area as well as stars highlight bands (or absence of bands) that indicate the "crack" spectra cannot be ascribed to neither the **1*i*** nor **1*f*** phase.

References Supporting Information:

[1] Bruker ACS, Madison, Wisconsin.

[2] G.M. Sheldrick, *Acta Cryst*. *A64*, **2008**, 112-122.

[3] A. L. Spek (2008) PLATON, A Multipurpose Crystallographic Tool, Utrecht University, Utrecht, The Netherlands

[4] A. L. Spek, *J. Appl. Cryst.* **2003**, 36, 7-13.

[5] Pawley, G. S. *J. Appl. Cryst*. **1981**, *14*, 357.

[6] Coelho, A. A. TOPAS-Academic, Version 4.1, 2007, see: http:// www.topas-academic.net.
